# Isolation, Identification, Anti-Inflammatory, and In Silico Analysis of New Lignans from the Resin of *Ferula sinkiangensis*

**DOI:** 10.3390/ph16101351

**Published:** 2023-09-25

**Authors:** Junchi Wang, Qi Zheng, Minghui Shi, Huaxiang Wang, Congzhao Fan, Guoping Wang, Yaqin Zhao, Jianyong Si

**Affiliations:** 1 The Key Laboratory of Bioactive Substances and Resources Utilization of Chinese Herbal Medicine, Ministry of Education, Institute of Medicinal Plant Development, Chinese Academy of Medical Sciences & Peking Union Medical College, Beijing 100193, China; jcwang@implad.ac.cn (J.W.); zhengqi@implad.ac.cn (Q.Z.); wanghuaxiang@implad.ac.cn (H.W.); 2 Xinjiang Institute of Chinese Materia Medica and Ethnodrug, Urumqi 830002, China; xjshmh@126.com (M.S.); fcz_840701@163.com (C.F.); ping112_003@163.com (G.W.); xjzyq123@126.com (Y.Z.)

**Keywords:** *Ferula sinkiangensis*, lignan, structural elucidation, ECD, anti-inflammatory, molecular docking

## Abstract

*Ferula sinkiangensis* K. M. Shen (Apiaceae) is distributed in arid desert areas of Xinjiang, and its resin is a traditional Chinese medicine to treat gastrointestinal digestive diseases. To explore bioactive components from *F. sinkiangensis*, three new lignans and thirteen known components were isolated. The structural elucidation of the components was established utilizing spectroscopic analyses together with ECD calculations. Griess reaction results indicated new compounds **1** and **2** significantly decreased NO production in LPS-stimulated RAW 264.7 macrophages, and ELISA results indicated that they effectively attenuated LPS-induced inflammation by inhibiting TNF-α, IL-1β, and IL-6 expressions. The in silico approach confirmed that compound **1** docked into the receptors with strong binding energies of −5.84~−10.79 kcal/mol. In addition, compound **6** inhibited the proliferation of AGS gastric cancer cells with IC_50_ values of 15.2 μM by suppressing the cell migration and invasion. This study disclosed that *F. sinkiangensis* might be a promising potential resource for bioactive components.

## 1. Introduction

Inflammation is the basis of many physiological and pathological processes. In cases of tissue stress or dysfunction, a similar adaptive response occurs, which primarily depends on tissue-resident macrophages. This response is characterized as being intermediate between the basal homeostatic state and a typical inflammatory response [1]. Inflammation-inducing pathogen-associated molecular patterns (PAMPs) include highly conserved structures, such as lipopolysaccharides (LPS), heat shock proteins (HSP), peptidoglycans (PGN) [2], and cytosine-phosphate-guanin motifs [3]. PAMPs are recognized by pattern recognition receptors (PRRs) [4]. Once they are activated, PRRs transduce signals intracellularly [5]. In addition, they induce, produce, and release proinflammatory mediators, including cytokines such as tumor necrosis factor alpha (TNF-*α*), interleukin (IL)-1, and IL-6 [6]. These proinflammatory cytokines are related to many diseases: TNF-α plays an important role in the occurrence of Crohn disease [7]; IL-1*β* is associated with sustained immune responses and contributes to a range of serious central nervous system (CNS) diseases, such as traumatic diabetic retinopathy, brain injury, neurodegenerative diseases, and multiple sclerosis [8]; IL-6 has been robustly associated with mortality outcomes in various conditions, including cancer, cardiovascular disease, and metabolic syndrome [9]. More and more evidence shows that inflammation is involved in the occurrence and development of common chronic diseases, such as cardiovascular disease, osteoporosis, diabetes, and cancer, etc. The application of anti-inflammatory treatment in these diseases is particularly important.

Clinically, steroidal anti-inflammatory drugs (SAIDs) and non-steroidal anti-inflammatory drugs (NSAIDs) are commonly used to treat inflammatory diseases. SAIDS usually refer to steroid hormones, including corticosteroids and glucocorticoids. These drugs have strong anti-inflammatory effects, but serious side effects such as water-sodium retention and infection are limited in clinical use [10]. NSAIDS are a class of anti-inflammatory drugs that do not contain steroidal structures, including aspirin, naproxen, diclofenac, ibuprofen, etc. They are well tolerated, but their long-term use is limited by severe gastrointestinal adverse reactions, and even gastrointestinal bleeding and perforation may occur in severe cases [11]. Therefore, the search for new anti-inflammatory drugs has been a research hotspot.

As the third-largest genus in the Apiaceae family, *Ferula* has approximately 180 species. *Ferula* is mainly distributed in the Mediterranean region and Asia, such as Saudi Arabia, Afghanistan, Turkey, Iran, India, and China, and has been used as remedies for various ailments worldwide [12]. Most *Ferula* plants have a pungent odor and bitter taste due to the presence of disulfides, such as 1-(methylthio)propyl (*E*)-1-propenyl disulfide, (*E*)-propenyl sec-butyl disulfide, foetisulfide A-D, etc. [13,14]. In Asia, they are also used as spices and as seasonings in kimchi, meat sauces, curry, and other foods [15]. The *Ferula* genus is mostly characterized by the presence of sesquiterpene coumarins [16], with a wide range of biological activities, including cytotoxic [17], anti-neuroinflammatory [18], antibacterial [19], antiviral [20], antioxidant [21], and α-glucosidase inhibition [22] properties.

*F. sinkiangensis* is an important member of this genus and its resin is recognized in the 2020 edition of *Chinese Pharmacopoeia*. As an endemic species in China, *F. sinkiangensis* is mainly distributed in arid desert areas of Xinjiang. *F. sinkiangensis* has a long history of local use, mainly for the treatment of stomach problems and rheumatoid arthritis [23]. Besides sesquiterpene coumarins [24,25,26], various metabolites such as coumarins, phenylpropanoids, lignans, steroidal esters, aromatic acids, sesquiterpenes, monoterpenes, benzofurans, and sulfanes have been identified from its roots, resin, seeds, and aerial parts [27,28,29,30]. Among these, lignans from *F. sinkiangensis* with anti-inflammatory properties [23,31] caught our attention. To search for new anti-inflammatory lignans, a pair of undescribed lignan diastereomers, one undescribed norlignan, together with thirteen known compounds have been isolated from the resin of *F. sinkiangensis*. Herein, the isolation, structural elucidation, anti-inflammatory activity, cytotoxicity, and in silico study of the isolated compounds are reported.

## 2. Results

### 2.1. Structural Elucidation

**Figure 1:**
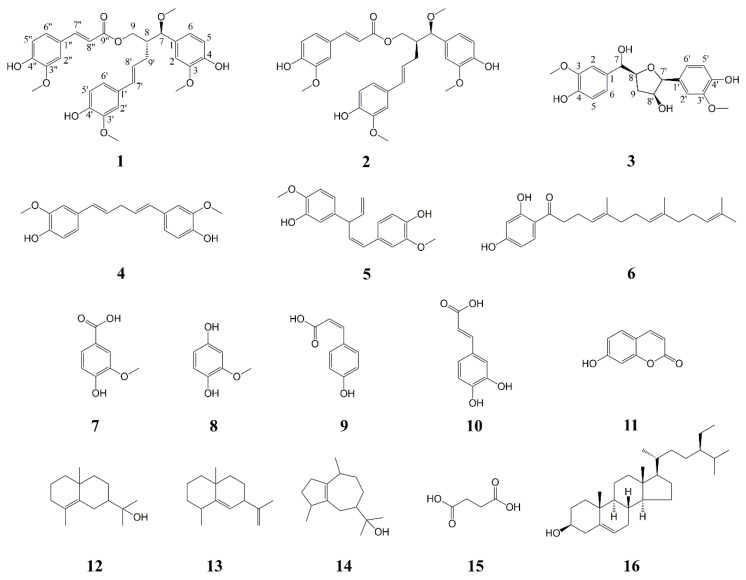
Compounds **1**–**16** isolated from *F. sinkiangensis*.

The phytochemical profile research of the resin of *F. sinkiangensis* gave a pair of new lignan diastereomers, (7*R*,8*R*)-sinkiangenone E and (7*R*,8*S*)-sinkiangenone E (**1**–**2**), one new norlignan, sinkiangenone F (**3**), together with thirteen known compounds, (1*E*,4*E*)-1-(4″-hydroxy-3″-methoxyphenyl)-5-(4′-hydroxy-3′-methoxyphenyl)-penta-1,4-dien (**4**) [32], (1*Z*,4*E*)-1-(3″-hydroxy-4″-methoxyphenyl)-3-(3’-hydroxy-4′-methoxyphenyl)-penta-1,4-dien (**5**) [33], dshamirone (**6**) [34], vanillic acid (**7**) [35], 2-methoxyhydroquinone (**8**) [36], (*Z*)-4-hydroxy cinnamic acid (**9**) [37], caffeic acid (**10**) [38], 7-hydroxycoumarin (**11**) [39], *γ*-eudesmol (**12**) [40], selina-5,11-diene (**13**) [41], guaiol (**14**) [42], succinic acid (**15**) [43], and stigmasterol (**16**) [44] (Figure 1).

Compound **1** was isolated as an orange gum. Its molecular formula was determined as C_31_H_34_O_9_ based on the HRESIMS data (*m*/*z* 573.2092 [M + Na]^+^, calcd for C_31_H_34_O_9_Na 573.2095), indicating fifteen indices of hydrogen deficiency. The IR absorption bands at 3369 and 1700 cm^−1^ were indicative of the presence of hydroxyl and carbonyl functional groups. The ^1^H NMR spectrum (Table 1) displayed the presence of three ABX spin system signals at *δ*_H_ 7.13 (1H, d, *J* = 1.8 Hz), 7.00 (1H, dd, *J* = 8.4, 1.8 Hz), 6.89 (1H, d, *J* = 1.8 Hz), 6.83 (1H, d, *J* = 1.8 Hz), 6.80 (1H, d, *J* = 8.4 Hz), 6.78 (1H, d, *J* = 8.4 Hz), 6.77 (1H, dd, *J* = 8.4, 1.8 Hz), 6.70 (1H, dd, *J* = 8.4, 1.8 Hz), and 6.64 (1H, d, *J* = 8.4 Hz), two *trans*-conformational olefinic signals at *δ*_H_ 7.53 (1H, d, *J* = 15.6 Hz), 6.33 (1H, d, *J* = 15.6 Hz), 6.16 (1H, d, *J* = 15.6 Hz), and 5.89 (1H, m), and four methoxy groups at *δ*_H_ 3.87, 3.83, 3.76, 3.17 (each 3H, s) (Table 1). The ^13^C-APT spectrum (Table 1) showed thirty-one well-resolved resonances (4 × CH_3_, 2 × CH_2_, 15 × CH, 10 × C) including a carbonyl carbon (*δ*_C_ 169.4), eighteen aromatic carbons, and four olefinic carbons (*δ*_C_ 150.6 to 110.0), an oxygenated methine carbon (*δ*_C_ 85.1), an oxygenated methylene carbon (*δ*_C_ 65.2), four methoxy carbons (*δ*_C_ 56.9, 56.4, 56.4 and 56.3), a methine carbon (*δ*_C_ 46.3), and a methylene carbon (*δ*_C_ 33.2). 

**Figure 2:**
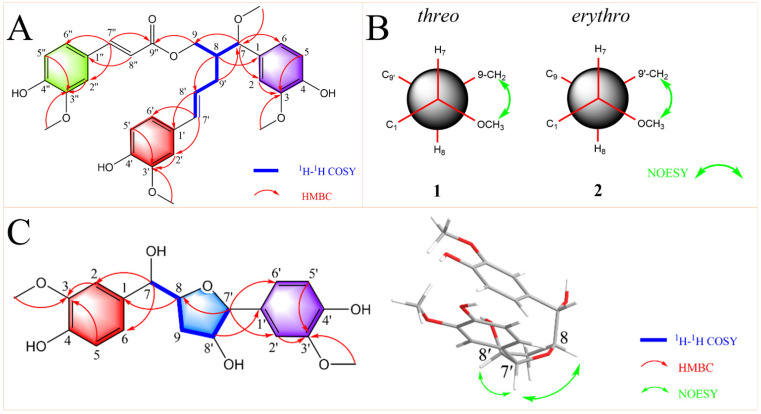
Key 2D correlations of compounds **1**–**3**. (**A**) Key ^1^H-^1^H COSY and HMBC correlations of compounds **1** and **2**. (**B**) Key NOESY correlations and relative configurations of compounds **1** and **2**. (**C**) Key ^1^H-^1^H COSY, HMBC, and NOESY correlations of compound **3**.

Comprehensive analysis of the HSQC and ^1^H-^1^H COSY data led to the identification of one partial structure (C-7-C-8(-C-9)-C-9′-C-8′-C-7′). In the HMBC spectrum (Figure 2A), the correlations from H-7″ to C-2″, C-6″, and C-9″; H-8″ to C-1″; H-9 to C-9″; H-8 to C-8′ and C-1; H-7 to C-2, C-,6 and C-9; H-9′ to C-7; H-8′ to C-1′; H-7′ to C-2′ and C-6′ established the skeleton structure of **1**. Additionally, the positions of four methoxy groups were also assigned by HMBC correlations from H-2, H-5, and -OCH_3_ (*δ*_H_ 3.76) to C-3; H-2′, H-5′ and -OCH_3_ (*δ*_H_ 3.83) to C-3′; H-2″, H-5″, and -OCH_3_ (*δ*_H_ 3.87) to C-3″; -OCH3 (*δ*_H_ 3.17) to C-7 (Figure 2A). Thus, the planar structure of compound **1** was established.

**Figure 3:**
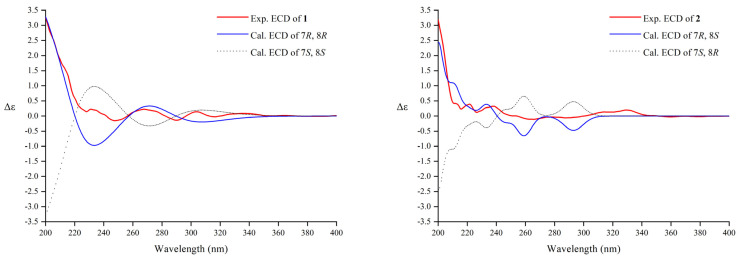
Comparison of experimental and calculated ECD spectra for compounds **1** and **2**.

According to reference [45], the relative configuration of the C-7-C-8 structural fragment of compound **1** was determined by a NMR configurational analysis based on coupling constants. In this case, the large ^3^*J*_H7-H8_ value (7.8 Hz) in the low-temperature NMR (−4 °C) [46] indicated two possible relative configurations (*threo* and *erythro*) because the two rotamers should have the opposite H/H orientation (Figure 2B). However, the coupling constant cannot distinguish the two conformers with opposite relative configurations; therefore, additional spatial information was required, typically dipolar effects. A strong correlation between H-9 and 7-OCH_3_ was observed in the NOESY spectrum of **1**, which indicated that C-9 and 7-OCH_3_ were close in three-dimensional space (Figure 2B). This dipolar coupling supported a *threo* stereochemical relationship (7*S*,8*S* or 7*R*,8*R*) for the pair of adjacent chiral carbons C-7 and C-8 to be assigned. Its absolute configuration was further determined by comparing experimental and calculated electrostatic circular dichroism (ECD) spectra because its single crystal cannot be obtained. Due to the great flexibility of the compound structure, we retrieved 2,239,488 conformations by Monte Carlo conformational search, and the conformers with a Boltzmann-population greater than 5% were selected for ECD calculations (Figure S10). Then, a steered molecular dynamics (SMD) polarization conductor calculation model in CH_3_OH was used for preliminary optimization by density functional theory (DFT) with the CAM-B3LYP/def2-SVP. At the CAM-B3LYP/def2-TZVP level, the theoretical calculation was conducted in CH_3_OH using time-dependent density functional theory (TD-DFT) for all conformers. As a result, the calculated ECD spectrum of an isomer (7*R*,8*R*) of compound **1** matched the experimental data (Figure 3). Thus, compound **1** was named (7*R*,8*R*)-sinkiangenone E.

Compound **2** was isolated as an orange gum. Its molecular formula was determined as C_31_H_34_O_9_ based on the HRESIMS data (*m*/*z* 573.2092 [M + Na]^+^, calcd for C_31_H_34_O_9_Na 573.2095). The IR, UV, and ^13^C NMR (Table 1) spectra of **2** were very similar to those of **1**, but in the ^1^H NMR spectrum (Table 1), the chemical shifts of H-9 (*δ*_H_ 4.09 and 3.90) and H-8 (*δ*_H_ 2.16) moved to the high-field area, while the chemical shifts of H-7 (*δ*_H_ 4.15), H-9′ (*δ*_H_ 2.60 and 2.39), H-8′ (*δ*_H_ 6.05), and H-7′ (*δ*_H_ 6.27) moved to the low-field area, compared to those of **1** (Figure S15), which suggested that the absolute configuration of **2** may be different from that of 1 at C-7 and C-8. The relative configuration of the C-7-C-8 segment of **2** was also confirmed by the same method [45]. The large ^3^*J*_H7-H8_ value (7.8 Hz) indicated the opposite H/H orientation (Figure 2B). In contrast to **1**, a strong correlation between H-9′ and 7-OCH_3_ was observed in the NOESY spectrum of **2**, indicating C-9′ and 7-OCH_3_ were close in three-dimensional space (Figure 2B). This dipolar coupling indicated an *erythro* stereochemical relationship (7*R*,8*S* or 7*S*,8*R*) for C-7 and C-8. By the same method (Figure 3), the absolute configuration of **2** was determined as 7*R*,8*S*. Compound **2** was then named (7*R*,8*S*)-sinkiangenone E.

Compound **3** was a colorless gum. According to HRESIMS data (*m*/*z* 385.1282 [M + Na]^+^, calcd for C_19_H_22_O_7_Na 385.1258), its molecular formula was determined to be C_19_H_22_O_7_, indicating that it has nine degrees of unsaturation. The IR absorption bands at 3297 cm^−1^ indicated the presence of a hydroxyl functional group. The ^1^H NMR spectrum (Table 1) displayed the presence of two ABX spin system signals at *δ*_H_ 6.97 (1H, d, *J* = 1.8 Hz), 6.94 (1H, d, *J* = 1.8 Hz), 6.81 (1H, dd, *J* = 8.4, 1.8 Hz), 6.78 (1H, dd, *J* = 8.4, 1.8 Hz), 6.73 (1H, d, *J* = 8.4 Hz), and 6.71 (1H, d, *J* = 8.4 Hz), and two methoxy groups at *δ*_H_ 3.81 and 3.80 (each 3H, s). The ^13^C-APT spectrum (Table 1) showed nineteen well-resolved resonances (2 × CH_3_, 1 × CH_2_, 10 × CH, 6 × C), including twelve aromatic carbons (*δ*_C_ 148.9 to 110.7), four oxygenated methine carbon (*δ*_C_ 89.9, 83.9, 79.3 and 78.1), two methoxy carbons (*δ*_C_ 56.4 and 56.3), and a methylene carbon (*δ*_C_ 37.7). 

The partial structure (C-7-C-8-C-9-C-8′-C-7′) was determined by a combined analysis of the HSQC and ^1^H-^1^H COSY data. Furthermore, the skeleton of **3** was established by the correlations from H-8 to C-1, H-7 to C-2 and C-6, H-8′ to C-1′, and H-7′ to C-8, C-2′ and C-6′ in the HMBC spectrum (Figure 2C). The two methoxy groups were also assigned through HMBC correlations from H-2, H-5, and -OCH_3_ (*δ*_H_ 3.80) to C-3, and H-2′, H-5′, and -OCH_3_ (*δ*_H_ 3.81) to C-3′ (Figure 2C). Finally, the two-dimensional structure of **3** was determined.

The relative configuration of the C-8-C-9-C-8′-C-7′-O segment of **3** was elucidated by the NOESY experiment (Figure 2C). In the NOESY spectrum, the correlations of H-8/H-7′/H-8′ indicated they were on the same side. Compound **3** was established as a norlignan and named sinkiangenone F.

### 2.2. Anti-Inflammatory Activity

**Figure 4:**
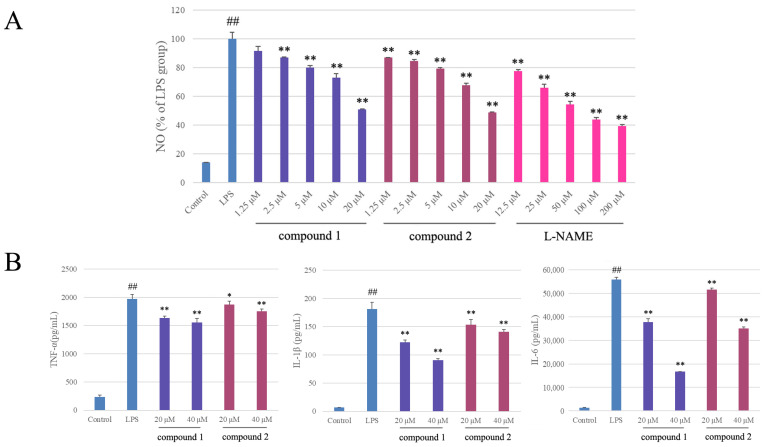
The anti-inflammatory effects of compounds **1** and **2** in LPS-induced RAW 264.7 cells. (**A**) Effects on NO production. (**B**) Effects on expressions of TNF-*α*, IL-1*β*, and IL-6. Each bar in the figures represents the mean ± SD of three independent experiments. Statistical differences were measured using ANOVA and Tukey’s post hoc test to identify significant differences between experimental groups. Significance: * *p* < 0.05 and ** *p* < 0.01 compared to LPS groups, ^##^ *p* < 0.001 compared to control groups.

The anti-inflammatory activities of the isolates were evaluated by the Griess assay in RAW 264.7 cells stimulated by LPS. Before that, the cytotoxicities of tested isolates were determined by the MTT method to avoid the potential impact of decreased viability on NO release. The results showed that compounds **1** and **2** significantly reduced NO production with IC_50_ values of 17.6 ± 0.4 μM and 14.9 ± 0.1 μM, respectively (cell viability > 90%), in a dose-dependent manner, while the positive control L-NAME was 47.4 ± 1.3 μM (Figure 4A). The other compounds did not show obvious anti-inflammatory activities (IC_50_ values > 50 μM).

TNF-*α*, IL-1*β*, and IL-6 are pro-inflammatory cytokines and important inflammatory mediators, which cause inflammation and related diseases. As shown in Figure 4B, LPS treatment significantly increased the expressions of TNF-*α*, IL-1*β*, and IL-6 from 237.0 ± 28.8, 6.8 ± 0.3, and 1362.2 ± 32.2 pg/mL to 1974.8 ± 79.6, 181.0 ± 12.2, and 55,923.2 ± 952.2 pg/mL, respectively, in RAW 264.7 macrophages. The expression levels of TNF-*α* were significantly decreased by pretreatment with compounds **1** and **2** (20 μM and 40 μM) to 1632.6 ± 33.5 and 1554.6 ± 70.4 pg/mL, and 1873.5 ± 57.8 and 1751.8 ± 41.9 pg/mL, respectively, compared to the positive control L-NAME (50 μM), 1158.7 ± 24.8 pg/mL. The expression levels of IL-1*β* were significantly decreased to 122.1 ± 4.3 and 90.4 ± 2.5 pg/mL, and 153.1 ± 9.9 and 140.8 ± 3.9 pg/mL, respectively, compared to L-NAME, 95.6 ± 4.1 pg/mL. The expression levels of IL-6 were significantly decreased to 37,809.3 ± 1338.7 and 16,695.5 ± 103.9 pg/mL, and 51,508.5 ± 635.6 and 34,972.0 ± 705.9 pg/mL, respectively, compared to L-NAME, 37,488.2 ± 1120.5 pg/mL. These results indicated that compounds **1** and **2** effectively attenuated LPS-induced inflammation by reducing the expressions of inflammatory molecules such as NO through inhibiting TNF-*α*, IL-1*β*, and IL-*6* expressions.

### 2.3. Molecular Docking Analysis

**Figure 5:**
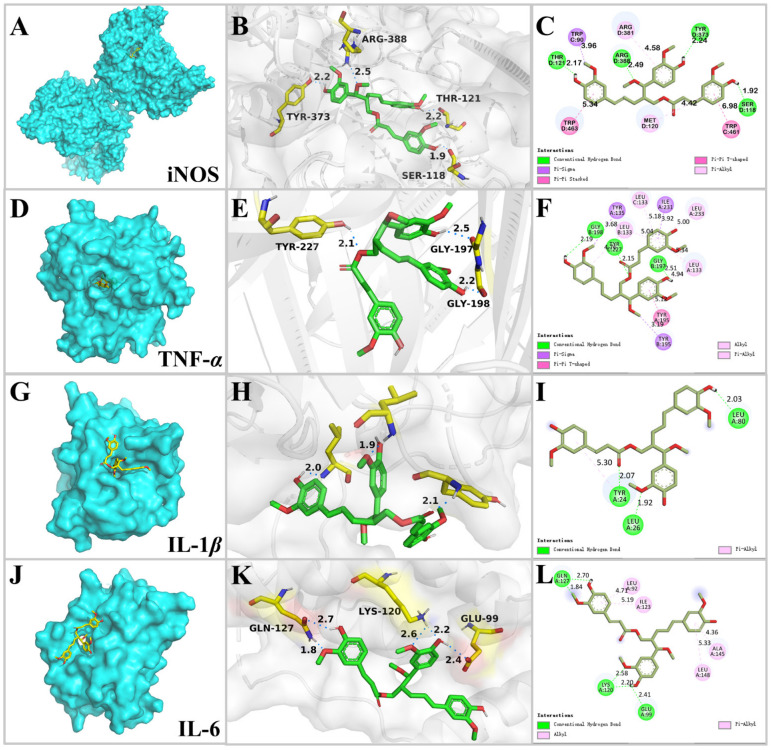
The binding mode of compound **1** with iNOS (PDB ID: 4CX7; (**A**–**C**)), TNF-α (PDB ID: 7JRA; (**D**–**F**)), IL-1β (PDB ID: 5R85; (**G**–**I**)), and IL-6 (PDB ID: 5FUC; (**J**–**L**)). Three-dimensional interactions with the protein surface to the left (visualized with PyMOL), 3D H-bond interactions in the middle (visualized with PyMOL), and 2D representation of the observed interactions to the right (visualized with Discovery Studio).

Through the anti-inflammatory experiments above, compound **1** showed strong inhibitory effects on NO production and TNF-α, IL-1β, and IL-*6* release. Therefore, molecular docking simulation was applied to predict the main binding patterns of compound **1** with inflammatory related proteins iNOS (inducible nitric oxide synthase, PDB ID: 4CX7), TNF-α (PDB ID: 7JRA), IL-1β (PDB ID: 5R85), and IL-6 (PDB ID: 5FUC) (Figure 5), to check whether the docking analyses supported the anti-inflammatory experimental results.

4CX7 is a complex of human iNOS with (*R*)-6-(3-amino-2-(5-(2-(6-amino-4-methylpyri-din-2-yl)ethyl)pyridin-3-yl)propyl)-4-methylpyridin-2-amine [47]. First, the co-crystallized ligand was re-docked with iNOS protein, and the dimensions of the grid box were determined based on the position of the co-crystallized ligand within the complex. The docking results indicated that the co-crystallized ligand formed three H-bonds with the residues of Arg 199, Glu 377, and Tyr 489 with a binding score of −10.0 kcal/mol. Then compound **1** was docked by the same procedure and the docking results revealed that compound **1** docked into the pocket of iNOS (Figure 5A) with a binding score of −9.42 kcal/mol. The ligand was stabilized by forming four H-bonds with the residues of Ser 118, Thr 121, Tyr 373, and Arg 388 (Figure 5B, Table 2). Additionally, the benzene rings and 3″-OCH_3_ of compound **1** formed hydrophobic interactions with the residues of Trp 90, Met 120, Arg 381, Trp 461, and Trp 463 (Figure 5C). L-NAME was selected as a control, which has been widely applied for several decades in both basic and clinical research as an antagonist of nitric oxide synthase (NOS) [48], and it was docked by the same procedure. The docking results revealed a binding score of −6.16 kcal/mol, which formed four H-bonds with the residues of Ala 262, Asn 283, Glu 285, and Glu 494. The binding score of compound **1** with iNOS was lower than that of L-NAME, indicating a strong binding interaction.

7JRA is a complex of human TNF-α with 2-[5-(3-chloro-4-{[(1*R*)-1-(2-fluorophenyl)ethyl]amino}quinolin-6-yl)pyrimidin-2-yl]propan-2-ol [49]. The co-crystallized ligand of 7JRA formed three H-bonds with the residues of Tyr 195 and Tyr 227 with a binding score of −10.94 kcal/mol. By the same procedure, compound **1** docked into the active site of TNF-α (Figure 5D) with a binding score of −10.79 kcal/mol. It formed three H-bonds including (1) the 4-OH and 4′-OH groups docked with the residues of Gly 197 and Gly 198 by serving as the H-bond donor, and (2) the oxygen of the ester group formed H-bond contact with the residue of Tyr 227 by performing as the H-bond acceptor. Additionally, the benzene rings, 7-OCH_3_, and 4′-OCH_3_ of compound **1** formed hydrophobic interactions with the residues of Leu 133, Tyr 135, Tyr 195, Ile231, and Leu 233 (Figure 5E,F). ZINC09609430 is a novel inhibitor for TNF-α [50], and its binding score was −9.64 kcal/mol, which was higher than that of compound **1**, by forming one H-bond with the residue of Tyr 227.

5R85 is a complex of IL-1β with Fragment Z1262246195 [51]. The co-crystallized ligand showed a binding score of −4.87 kcal/mol by forming three H-bonds with the residues of Leu 26, Leu 82, and Val 132. Compound **1**-IL-1β complex (Figure 5G) exhibited a lower binding score of −7.81 kcal/mol. It formed three H-bonds with the residues of Tyr 24, Leu 26, and Leu 80. Additionally, compound **1** was stabilized by hydrophobic interaction with the residues of Tyr 24 (Figure 5H,I). Aequilabrines C is a new lignan isolated from *Justicia aequilabris* with a maximum inhibition rate of IL-1β production 32.5% at 16.7 μM [52]. The docking results revealed that the binding score of aequilabrines C with IL-1β was −7.26 kcal/mol, by forming two H-bonds with the residues of Glu 25 and Leu 134. 

5FUC is a complex of IL-6 and gp80 [53]. Due to the absence of a co-crystallized small-molecule ligand in 5FUC, the grid box was set to cover the surface of IL-6. The compound **1**-IL-6 complex (Figure 5J) showed a binding score of −5.84 kcal/mol; specifically, compound **1** showed interactions with Glu 99, Lys 120, and Gln 127 through five H-bonds. Compound **1** also displayed hydrophobic interactions with Leu 92, Ile 123, Ala 145, and Leu 148 (Figure 5K,L). LMT 28 was selected as a control because it is a novel synthetic IL-6 inhibitor [54]. The docking results revealed a binding score of −5.21 kcal/mol, which was higher than that of compound **1**, by forming two H-bonds with the residue of Asp 140.

### 2.4. Cytotoxicity

To evaluate their cytotoxic activities, all the isolates (**1**–**16**) were tested in vitro against three human cancer cell lines (MGC-803, AGS, and HeLa), using the MTT viability assay with Taxol as a positive control. Compound **6** inhibited the proliferation of MGC-803, AGS, and HeLa cells with moderate cytotoxicity (Table 3), with IC_50_ values ranging from 15.2 to 30.5 μM, while compounds **1** and **2** displayed weak cytotoxic activities against AGS and HeLa (IC_50_ 41.9–63.0 μM).

### 2.5. Compound ***6*** Inhibits the Migration and Invasion of Gastric Cancer Cells

**Figure 6:**
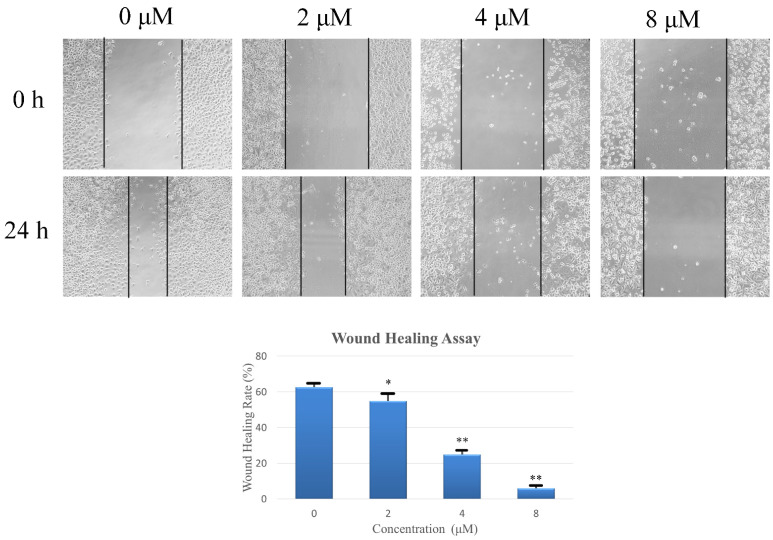
The wound healing assay. A linear wound was created, and the cells were treated for 24 h with various concentrations of compound **6** (0, 2, 4, or 8 μM), diluted using serum-free RPMI-1640 medium. Images of the wounds were taken at 0 and 24 h using an inverted microscope with 100× magnification. The experiments were performed in triplicate, and the statistical results are expressed as means ± SD. Statistical significance (* *p* < 0.05 and ** *p* < 0.01) was determined by comparing the treatment groups to the control group.

Cell migration and invasion are crucial processes involved in cancer metastasis. The effects of compound **6** on motility in AGS cells were examined through a wound healing experiment, and low cytotoxicity concentrations of compound **6** were selected to avoid any interference from cytotoxicity-related effects. As shown in Figure 6, the wound healing rate in MGC803 cells displayed an inverse relationship with compound **6** concentration. After 24 h, the wound healing rate was 62.67% in the control group, which decreased to 24.94% in the 4 μM compound **6**-treatment group. Notably, the wound healing rates dropped to less than 10.00% upon treatment with 8 μM of compound **6** for 24 h.

**Figure 7:**
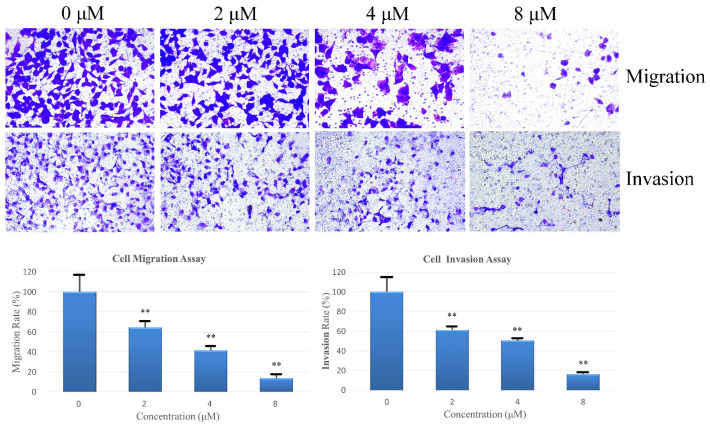
Compound **6** inhibited the migration and invasion of AGS cells. Images of the cells were captured using an inverted microscope with 200× magnification. The number of cells invading through the transwell chambers was counted, and the migration and invasion potentials were quantified for each treatment group. The experiments were performed in triplicate, and the statistical results are expressed as means ± SD. Statistical significance (** *p* < 0.01) was determined by comparing the treatment groups to the control group.

Following this, the transwell chamber assay was carried out using the same concentration gradient to further examine the inhibitory effects of compound **6** on AGS cell line migration and invasion. The results are presented in Figure 7, showing compound **6** exhibited inhibitory effects on AGS cell line migration and invasion. At a concentration of 8 μM, the migration and invasion rates were significantly reduced to 13.74% and 16.28%, respectively, compared to those of the control group.

## 3. Discussion

A lignan is a kind of secondary metabolite of plants formed by the oxidative polymerization of two phenylpropanoid units, and its biological activity is closely related to its structural diversity. So far, natural products of lignans have been found to have many promising biological activities, including anti-inflammatory, anti-menopausal, antibacterial, antiviral, antitumor, antiplatelet, phosphodiesterase inhibition, 5-lipoxygenase inhibition, HIV reverse transcription inhibition, cytotoxic, antioxidant, immunosuppressive, and antiasthmatic activities [55,56]. Various molecular mechanisms contribute to the pro-inflammatory process. The NF-κB and MAPK pathways can be activated through phosphorylation and translocation of NF-κB and AP-1 subunits, which upregulate pro-inflammatory cytokines, including iNOS, TNF-*α*, IL-1*β*, and IL-6 [57,58], and it has been reported that Schisandrin B, the lignan found in *Schisandra chinensis*, exerted anti-inflammatory effects by modulating these pathways [59]. In brief, studies have shown that lignans are potential therapeutic agents in inflammatory conditions. 

In this study, a pair of new lignan diastereomers, (7*R*,8*R*)-sinkiangenone E and (7*R*,8*S*)-sinkiangenone E (**1**–**2**), one new norlignan, sinkiangenone F (**3**), together with thirteen known compounds (**4**–**16**), were isolated from the resin of *F. sinkiangensis*. The structures of the new compounds were elucidated based on a combined analysis of the IR, UV, HRESIMS, NMR, and ECD spectra. Compounds **1** and **2** were diastereomers, their relative configurations were identified through a *J*-based NMR configurational analysis coupled with NOESY spectra, and their absolute configurations were further determined by a comparison of calculated ECD curves with experimental ECD curves. The flexibility of the compound structure makes it difficult to calculate ECD curves; therefore, we used the basis set of def2-TZVP, which is the ideal basis set for high-precision DFT calculation [60]. Griess reaction and ELISA results indicated that compounds **1** and **2** effectively attenuated LPS-induced inflammation by reducing the production of inflammatory molecules such as NO through inhibiting TNF-*α*, IL-1*β*, and IL-6 expressions. An in silico approach was used to verify the anti-inflammatory properties of compound **1** by determining the binding scores of compound **1** with iNOS, TNF-*α*, IL-1*β*, and IL-6, and known compounds previously reported as targeting the same proteins, such as L-NAME, ZINC09609430, aequilabrines C, and LMT-28, were selected as controls. The binding scores of compound **1** with established targets were lower than those of comparative compounds, indicating strong binding interactions with iNOS, TNF-*α*, IL-1*β*, and IL-6 proteins. These docking analyses supported the experimental results of the above Griess assay and ELISA on compound **1**. This study disclosed that *F. sinkiangensis* might be a promising potential resource for the discovery of new anti-inflammatory compounds.

## 4. Materials and Methods

### 4.1. Experimental

HRESIMS data were acquired using a Thermo Scientific LTQ-Orbitrap XL instrument (Bremen, Germany). The IR spectra data were recorded using a Shimadzu FTIR-8400S spectrometer (Kyoto, Japan). The UV spectra data were recorded using a Shimadzu UV-2550 spectrophotometer (Kyoto, Japan). The optical rotation data were determined at 25 °C in CH_3_OH using a Perkin-Elmer 341 digital polarimeter (Waltham, MA, USA). NMR spectra data were recorded on a Bruker AV III 600 NMR spectrometer (Rheinstetten, Germany). Column chromatography was performed using Sephadex LH-20 (Pharmacia Biotech, Stockholm, Sweden) and silica gel (200–300 mesh). Semipreparative HPLC was conducted on a Chuangxintongheng K1001 analytic LC instrument with an Agilent-SB-Phenyl (250 × 10 mm, 5 μm) and a YMC-C18 column (250 × 10 mm, 5 μm). Penicillin, streptomycin, trypsin, MTT, DMEM, and FBS were purchased from Gibco (Carlsbad, CA, USA). DMSO, L-NAME, and LPS were purchased from Sigma-Aldrich (St. Louis, MO, USA). TNF-*α*, IL-1*β*, and IL-6 ELISA kits were purchased from Biolegend (San Diego, CA, USA).

### 4.2. Material

The *F. sinkiangensis* resin was collected from Yili state, Xinjiang Uygur Autonomous Region, China, and authenticated by Prof. Congzhao Fan (Xinjiang Institute of Chinese Materia Medica and Ethnodrug, Urumqi, China). The voucher specimen (FSR-202106) was deposited at the Institute of Medicinal Plant Development, Chinese Academy of Medical Sciences & Peking Union Medical College, Beijing, China.

### 4.3. Extraction and Isolation

The *F. sinkiangensis* resin (1.4 kg) was under reflux extraction by 95% ethanol. Then, a crude extract (1000 g) was obtained by removing the solvent. After that, extracts from four parts were acquired by suspending the crude extract in water and extracting with petroleum ether (PET), dichloromethane, ethyl acetate, and n-butanol, respectively. 

The dichloromethane extract (700 g) was then chromatographed with CH_2_Cl_2_- CH_3_OH (1:0~0:1, *v*/*v*) by silica gel to obtain seven fractions (Fr. 1~Fr. 7). Fr. 1 was chromatographed with PET-EtOAc (1:0~0:1, *v*/*v*) to afford five fractions (Fr. 1-1~Fr. 1-5). Fr. 1-3 was then chromatographed with PET-EtOAc (50:0~0:1, *v*/*v*) to afford **12** (88 mg). Fr. 1-4 was chromatographed with PET-EtOAc (60:1, *v*/*v*) to afford **14** (26 mg). Fr. 2 was chromatographed by silica gel with PET-EtOAc (30:1~0:1, *v*/*v*) to obtain four fractions (Fr. 2-1~Fr. 2-4). Fr. 2-1 was purified with PET to afford **13** (36 mg). Fr. 2-2 was chromatographed by semipreparative HPLC with a YMC-C18 column (CH_3_OH -H_2_O, 98%:2%, 17.8 min), to afford **6** (12 mg). Fr. 3 was chromatographed over silica gel with n-hexane-acetone (20:1~0:1, *v*/*v*) to afford seven fractions (Fr. 3-1~Fr. 3-7). Fr. 3-2 was purified with PET-EtOAc (40:1~0:1, *v*/*v*) to afford **16** (5 mg). Fr. 3-5 was purified with CH_2_Cl_2_-CH_3_OH (60:1~0:1, *v*/*v*) to give nine fractions (Fr. 3-5-1~Fr. 3-5-9). Fr. 3-5-1 was recrystallized to obtain **11** (25 mg). Fr. 3-5-3 was chromatographed by semipreparative HPLC with an Agilent-SB-Phenyl column (CH_3_OH-H_2_O, 70%:30%, 13.0 min), to afford **5** (14 mg). Fr. 4 was chromatographed over silica gel with CH_2_Cl_2_-CH_3_OH (1:0~0:1, *v*/*v*) to give five fractions (Fr. 4-1~Fr. 4-5). Fr. 4-2 was purified by a preparative TLC method (CHCl_3_-CH_3_OH- H_2_O, 8.5:1.5:1, *v*/*v*/*v*) to obtain **9** (15 mg). Fr. 6 was separated over MCI gel with CH_3_OH-H_2_O (60%:40%~100:0, *v*/*v*) to give four fractions (Fr. 6-1~Fr. 6-4). Fr. 6-3 was chromatographed on a Sephadex LH-20 column to afford three fractions (Fr. 6-3-1~Fr. 6-3-3). Fr. 6-3-2 was purified by semipreparative HPLC with an Agilent-SB-Phenyl column (CH_3_OH-H_2_O, 75%:25%), to afford **1** (17.2 min, 7 mg) and **2** (18.7 min, 5 mg). Fr. 6-3-3 was purified by semipreparative HPLC with an Agilent-SB-Phenyl column (CH_3_OH-H_2_O, 80%:20%, 11.1 min), to afford **4** (10 mg).

The n-butanol part (21 g) was chromatographed through MCI using CH_3_OH-H_2_O elution (40%:60%~100%:0, *v*/*v*) to acquire Fr. 1~Fr. 9, of which Fr. 2 was further chromatographed by Sephadex LH-20 to acquire Fr. 2-1~Fr. 2-3. Fr. 2-2 was chromatographed by semipreparative HPLC with an Agilent-SB-Phenyl column (CH_3_OH-H_2_O, 35%:65%, 17.5 min), to afford **3** (4 mg). Fr. 2-3 was chromatographed by semipreparative HPLC with an Agilent-SB-Phenyl column (CH_3_OH-H_2_O, 48%:52%, 11.7 min), to afford **10** (2 mg). Fr. 3 was chromatographed by semipreparative HPLC with an Agilent-SB-Phenyl column (CH_3_OH-H_2_O, 45%:65%, 12 min), to afford **8** (4 mg). Fr. 4 was chromatographed by semipreparative HPLC with an Agilent-SB-Phenyl column (CH_3_OH-H_2_O, 60%:40%, 10.3 min), to afford **7** (2 mg). Fr. 5 was chromatographed by semipreparative HPLC with an Agilent-SB-Phenyl column (CH_3_OH-H_2_O, 60%:40%, 10.3 min), to afford **15** (2 mg).

(7*R*,8*R*)-Sinkiangenone E (**1**): Orange gum; UV (CH_3_OH) λ_max_ (log ε): 318 (4.64) nm; [α${]}_{D}^{25}$: −17.9 (c = 0.084, CH_3_OH); IR: 3369, 2935, 1700, 1597, 1515, 1271, 1158, 1124, 1031 cm^−1^; ^1^H NMR (600 MHz, CD_3_OD) and ^13^C NMR (150 MHz, CD_3_OD) data, see Table 1; HRESIMS (positive mode) *m*/*z*: 573.2092 [M + Na]^+^ (calcd for C_31_H_34_O_9_Na, 573.2095).

(7*R*,8*S*)-Sinkiangenone E (**2**): Orange gum; UV (CH_3_OH) λ_max_ (log ε): 322 (4.64) nm; [α${]}_{D}^{25}$: +15.0 (c = 0.04, CH_3_OH); IR: 3437, 2936, 1696, 1596, 1507, 1272, 1158, 1123, 1033 cm^−1^; ^1^H NMR (600 MHz, CD_3_OD) and ^13^C NMR (150 MHz, CD_3_OD) data, see Table 1; HRESIMS (positive mode) *m*/*z*: 573.2092 [M + Na]^+^ (calcd for C_31_H_34_O_9_Na, 573.2095).

Sinkiangenone F (**3**): Colorless gum; UV (CH_3_OH) λ_max_ (log ε): 229 (4.07), 279 (3.70) nm; [α${]}_{D}^{25}$: +0.7 (c = 0.135, CH_3_OH); IR: 3297, 2936, 2920, 1653, 1635, 1558, 1521, 1506, 1272, 1032 cm^−1^; ^1^H NMR (600 MHz, CD_3_OD) and ^13^C NMR (150 MHz, CD_3_OD) data, see Table 1; HRESIMS (positive mode) *m*/*z*: 385.1282 [M + Na]^+^ (calcd for C_19_H_22_O_7_Na, 385.1258).

### 4.4. ECD Simulation

Spartan’s 14 v1.1.4 software was applied to perform the Monte Carlo conformational search, using the method of Merck Molecular Force Field (MMFF). At the CAM-B3LYP/def2-SVP level, the conformers of compounds **1** and **2** with a Boltzmann population greater than 5% were selected for optimization in CH_3_OH. Then, an SMD polarization conductor calculation model was used for excited state calculations of the CAM-B3LYP/def2-TZVP level in CH_3_OH, employing TD-DFT for all conformers. A total of 60 excited states were calculated to determine their rotatory strengths. ECD spectra were generated from dipole-length rotational strengths using SpecDis 1.71 and Origin 2019b, applying Gaussian band shapes with a sigma of 0.3 eV and 0.12 eV.

### 4.5. Determination of NO Production by the Griess Assay

RAW 264.7 cells were purchased from the Institute of Basic Medical Sciences, Chinese Academy of Medical Sciences (Beijing, China). MTT assay was applied to assess cell viability [61]. The macrophages were cultured in DMEM with 10% FBS. After 24 h of incubation, 100 μL of tested compounds were added. After 24 h, 20 μL of MTT was added into each well. After another 4 h, the supernatant was discarded, and 150 μL of DMSO was added into each well. After shaking for 1 min, the optical density of each well was detected at 570 nm wavelength by a microplate reader (Sunrise, Grödig, Austria), and the cell viability was calculated.

The Griess reaction was applied to evaluate the inhibitory effects of isolated compounds on NO production in RAW 264.7 cells induced by LPS [62]. The cells were seeded in 96-well plates with DMEM containing 10% FBS, together with LPS (100 ng/mL) and L-NAME (positive control) or isolated compounds. After that, 50 μL of culture supernatant and 50 μL of Griess reagent were mixed at room temperature. After another 15 min, the absorbance at 540 nm was detected.

### 4.6. Enzyme-Linked Immunosorbent Assay (ELISA)

RAW 264.7 macrophages were treated with LPS (100 ng/mL) and isolated compounds for 24 h. Then, the culture medium was collected and used for measuring the expression levels of respective proinflammatory cytokines (TNF-*α*, IL-1*β*, and IL-6). The experiments were conducted using ELISA kits following the manufacturer’s protocols.

### 4.7. Molecular Docking

Molecular docking was performed to check whether the in silico study supported the anti-inflammatory experimental results. The X-ray crystal structures of iNOS (PDB ID: 4CX7), TNF-α (PDB ID: 7JRA), IL-1β (PDB ID: 5R85), and IL-6 (PDB ID: 5FUC) proteins were obtained from RCSB Protein Data Bank (https://www.rcsb.org/, accessed on 7 September 2023). Ligands (compound **1**, L-NAME, ZINC09609430, aequilabrines C, and LMT-28) were drawn in ChemBioDraw Ultra 14.0 and were copied to ChemBio3D Ultra 14.0; then, MM2 calculation was used to minimize the energy of conformation and structures were saved in PDB format. Before docking, water molecules and any other unwanted components (including co-crystallized small-molecule ligands of 4CX7, 7JRA, and 5R85, and gp80 protein of 5FUC) were removed from proteins and ligands. The proteins were prepared by adding polar hydrogen atoms and assigning partial charges, and the ligand was prepared by assigning atom types, charges, and torsion angles. Then, the search spaces or active sites on the proteins were defined by the co-crystallized ligands, and grid boxes (grid dimensions of 4CX7: 70 Å × 70 Å × 72 Å; grid dimensions of 7JRA: 46 Å × 42 Å × 50 Å; grid dimensions of 5R85: 34 Å × 36 Å × 34 Å; grid dimensions of 5FUC: 122 Å × 90 Å × 98 Å) were generated within the specified search. After that, AutoDock was used to perform the molecular docking calculation and to predict the binding affinity of the ligand to the proteins. Docking parameters were set to 100 genetic algorithm runs and ligands were docked via the Lamarckian GA. The results of molecular docking were visualized by PyMOL Molecular Graphics System v.2.2.0 and Discovery Studio 45.

### 4.8. Cytotoxicity 

MGC-803, AGS, and HeLa cell lines were also purchased from the Institute of Basic Medical Sciences, Chinese Academy of Medical Sciences (Beijing, China). The cytotoxicities of the isolates were evaluated using a modified MTT assay [63]. AGS and HeLa cells were cultured in RPMI 1640 medium, while MGC-803 cells were cultured in DMEM, supplemented with 10% FBS. MGC-803, AGS, and HeLa cells were incubated into 96-well plates with the concentration of 3 × 10^4^, 7 × 10^4^, and 5 × 10^4^ cells per mL, respectively. After 24 h of incubation, 100 μL of tested compounds of different concentrations were added, and DMSO was used as the solvent control. After 48 h, 20 μL of MTT was added into each well. After another 4 h, the supernatant was discarded, and 150 μL of DMSO was added into each well. After shaking for 1 min, the optical density of each well was detected at 570 nm wavelength by a microplate reader (Sunrise, Austria), and the cell viability was calculated.

### 4.9. Wound Healing Assay

The experimental procedure involved culturing cells in the logarithmic phase until approximately 90% confluence was achieved in a 24-well plate. In the monolayer of cells, a sterile 200 μL pipette tip was used to create a linear wound. After rinsing with PBS to remove unattached cells and debris, the AGS cells were treated with compound **6** (0, 2, 4, or 8 μM) for 24 h. At 0 and 24 h, wound images were taken by an inverted microscope at 100× magnification. Then, Image J 1.53a software was used to determine the wound area. Finally, the change in wound area was used to calculate the wound healing rate.

### 4.10. Cell Migration and Invasion Assays

Transwell chambers were used with or without Matrigel. In migration experiments, cells (3 × 10^4^ each well) were added directly without any coating. In invasion experiments, the cells were pre-coated with a layer of Matrigel. The lower chamber wells contained 600 μL of RPMI-1640 medium supplemented with 10% FBS and compound **6** (0, 4, 8, or 16 μM). The Transwell chambers were then incubated for 24 h, methanol was used to fix the cells for 20 min, crystal violet was used to stain the cells for 30 min, and an inverted microscope was used to image at 200× magnification. The numbers of cells that had migrated or invaded were counted and used to calculate migration or invasion rates.

### 4.11. Statistical Analysis

SPSS 22.0 software was applied to perform statistical analysis. The results were expressed as mean ± standard deviation. The significance between each group was * *p* < 0.05, ** *p*< 0.01, and ^##^ *p* < 0.001. 

## 5. Conclusions

In summary, three new lignans and thirteen known compounds were isolated from the resin of *F. sinkiangensis*. Compounds **1** and **2** were diastereomers; their relative configurations were identified through a *J*-based NMR configurational analysis coupled with NOESY spectra, and their absolute configurations were further determined by a comparison of calculated ECD curves with experimental ECD curves. Griess reaction and ELISA results indicated that compounds **1** and **2** effectively attenuated LPS-induced inflammation by reducing the production of NO and TNF-α, IL-1β, and IL-6 expressions. Molecular docking results showed that the binding scores of compound **1** with established targets were lower than those of comparative compounds, indicating strong binding interactions with iNOS, TNF-α, IL-1β, and IL-6 proteins. Compound **6** exhibited potential cytotoxicity to AGS gastric cancer cells by suppressing cell migration and invasion. All these results indicated that *F. sinkiangensis* might be a promising potential resource for the discovery of new anti-inflammatory lignans.

## Figures and Tables

**Table 1:** ^1^H (600 MHz) and ^13^C (150 MHz) NMR data for compounds **1**–**3** (CD_3_OD).

No.	Compound 1		Compound 2		Compound 3	
	*δ*_C_	*δ*_H_ (*J* in Hz)	*δ*_C_	*δ*_H_ (*J* in Hz)	*δ*_C_	*δ*_H_ (*J* in Hz)
1	132.6	-	132.7	-	134.2	-
2	111.7	6.89 (d, 1.8)	111.6	6.86 (d, 1.8)	111.7	6.97 (d, 1.8)
3	147.0	-	147.0	-	148.9	-
4	149.0	-	149.0	-	147.3	-
5	116.1	6.64 (d, 8.4)	116.1	6.66 (d, 8.4)	115.8	6.71 (d, 8.4)
6	121.7	6.77 (dd, 8.4, 1.8)	121.2	6.74 (dd, 8.4, 1.8)	121.0	6.81 (dd, 8.4, 1.8)
7	85.1	4.11 (d, 7.8)	85.2	4.15 (d, 7.8)	78.1	4.56 (d, 6.6)
8	46.3	2.19 (m)	46.4	2.16 (m)	83.9	4.35 (dt, 9.0, 6.6)
9	65.2	4.35 (d, 4.8)	65.4	4.09 (dd, 10.8, 4.8) 3.90 (dd, 10.8, 4.8)	37.7	1.86 (ddd, 13.2, 9.0, 6.6) 1.51 (ddd, 13.2, 6.0, 3.0)
1′	131.3	-	131.4	-	133.9	-
2′	110.0	6.83 (d, 1.8)	110.1	6.89 (d, 1.8)	110.7	6.94 (d, 1.8)
3′	147.4	-	147.3	-	148.9	-
4′	149.2	-	149.2	-	147.0	-
5′	116.0	6.80 (d, 8.4)	116.1	6.78 (d, 8.4)	115.9	6.73 (d, 8.4)
6′	120.4	6.70 (dd, 8.4, 1.8)	120.4	6.76 (dd, 8.4, 1.8)	119.8	6.78 (dd, 8.4, 1.8)
7′	132.9	6.16 (d, 15.6)	133.0	6.27 (d, 15.6)	89.9	4.64 (d, 3.6)
8′	126.2	5.89 (m)	126.4	6.05 (m)	79.3	3.98 (dt, 6.0, 3.6)
9′	33.2	2.16 (m), 2.10 (m)	32.7	2.60 (m), 2.39 (m)		
1″	127.6	-	127.6	-		
2″	111.6	7.13 (d, 1.8)	111.5	7.12 (d, 1.8)		
3″	149.3	-	149.4	-		
4″	150.6	-	150.7	-		
5″	116.4	6.78 (d, 8.4)	116.4	6.75 (d, 8.4)		
6″	124.2	7.00 (dd, 8.4, 1.8)	124.2	6.98 (dd, 8.4, 1.8)		
7″	115.5	6.33 (d, 15.6)	115.4	6.30 (d, 15.6)		
8″	146.8	7.53 (d, 15.6)	146.8	7.48 (d, 15.6)		
9″	169.4	-	169.2	-		
3-OCH_3_	56.3	3.76 (s)	56.3	3.77 (s)	56.3	3.80 (s)
7-OCH_3_	56.9	3.17 (s)	57.1	3.21 (s)		
3′-OCH_3_	56.4	3.83 (s)	56.3	3.80 (s)	56.4	3.81 (s)
3″-OCH_3_	56.4	3.87 (s)	56.4	3.87 (s)		

**Table 2:** Calculated docking affinities of compound **1** to iNOS, TNF-α, IL-1β, and IL-6 proteins by AutoDock.

Protein	Affinity Score (Kcal/mol)	Conventional Hydrogen Bond	Hydrophobic Interactions
iNOS (PDB ID: 4CX7)	−9.42	Ser 118, Thr 121, Tyr 373, Arg 388	Trp 90, Met 120, Arg 381, Trp 461, Trp 463
TNF-α (PDB ID: 7JRA)	−10.79	Gly 197, Gly 198, Tyr 227	Leu 133, Tyr 135, Tyr 195, Ile231, Leu 233
IL-1β (PDB ID: 5R85)	−7.81	Tyr 24, Leu 26, Leu 80	Tyr 24
IL-6 (PDB ID: 5FUC)	−5.84	Glu 99, Lys 120, Gln 127	Leu 92, Ile 123, Ala 145, Leu 148

**Table 3:** Cytotoxicity of compounds **1–16** (IC_50_^a^, μM).

	MGC-803	AGS	HeLa
**1**	>100	63.0 ± 1.8	41.9 ± 1.7
**2**	>100	61.5 ± 1.5	45.9 ± 0.6
**3**	>100	>100	>100
**4**	>100	>100	>100
**5**	>100	>100	>100
**6**	30.5 ± 1.3	15.2 ± 0.9	30.2 ± 0.4
**7**	>100	>100	>100
**8**	>100	>100	>100
**9**	>100	>100	>100
**10**	>100	>100	>100
**11**	>100	>100	>100
**12**	47.9 ± 1.8	>100	92.0 ± 12.3
**13**	>100	>100	>100
**14**	>100	>100	>100
**15**	>100	>100	>100
**16**	>100	>100	>100
Taxol ^b^	3.4 ± 0.1	1.8 ± 0.1	7.5 ± 0.4

^a^ Values are expressed as the means ± SD from three independent experiments. ^b^ Positive control.

## Data Availability

The data presented in this study are available on request from the corresponding author.

## Supplementary Materials

The following supporting information can be downloaded at https://www.mdpi.com/article/10.3390/ph16101351/s1: Figures S1–S29: HRESIMS, UV, IR, ^1^H, ^13^C, ^1^H-^1^H COSY, HSQC, HMBC and NOESY NMR spectra of **1**–**3**.
