# Supplementary material for: Isolation, Identification, Anti-Inflammatory, and In Silico Analysis of New Lignans from the Resin of Ferula sinkiangensis
Source: Pharmaceuticals (Basel). 2023 Sep 25;16(10):1351. doi: 10.3390/ph16101351 (PMC10610263; doi:10.3390/ph16101351)
Supplement: Supplementary file 1 [file pharmaceuticals-16-01351-s001.zip › pharmaceuticals-2602214-supplementary.pdf]

Isolation, identification, anti-inflammatory, and *in silico* analysis of new lignans from *Ferula sinkiangensis*

Junchi Wang [1], Qi Zheng [1], Minghui Shi [2], Huaxiang Wang [1], Congzhao Fan [2], Guoping Wang [2], Yaqin Zhao [2], Jianyong Si [1,*]

[1] The Key Laboratory of Bioactive Substances and Resources Utilization of Chinese Herbal Medicine, Ministry of Education, Institute of Medicinal Plant Development, Chinese Academy of Medical Sciences & Peking Union Medical College, Beijing 100193, China; jcwang@implad.ac.cn (J.W.); zhengqi@implad.ac.cn (Q.Z.); wanghuaxiang@implad.ac.cn (H.W.)

[2] Xinjiang Institute of Chinese Materia Medica and Ethnodrug, Urumqi, 830002, China; xjshmh@126.com (M.S.); fcz_840701@163.com (C.F.); ping112_003@163.com (G.W.); xjzyq123@126.com (Y.Z.)

[*] Correspondence: jysi@implad.ac.cn (J.S.); Tel.: +86-010-57833299

Contents

MS, UV, IR and NMR Spectra of Compound 1

Fig. S1. HRESIMS spectrum of **1**

Fig. S2. IR spectrum of **1**

Fig. S3. UV spectrum in MeOH of **1**

Fig. S4. ^{1}H-NMR spectrum (CD_3OD, 600 MHz) of **1**

Fig. S5. ^{13}C-NMR spectrum (CD_3OD, 150 MHz) of **1**

Fig. S6. ^{1}H-^{1}H COSY spectrum (CD_3OD) of **1**

Fig. S7. HSQC spectrum (CD_3OD) of **1**

Fig. S8. HMBC spectrum (CD_3OD) of **1**

Fig. S9. NOESY spectrum (CD_3OD) of **1**

Fig. S10 The conformers of compounds **1** and **2** with a Boltzmann-population greater than 5% selected for ECD calculations

MS, UV, IR and NMR Spectra of Compound 2

Fig. S11. HRESIMS spectrum of **2**

Fig. S12. IR spectrum of **2**

Fig. S13. UV spectrum in MeOH of **2**

Fig. S14. ^{1}H-NMR spectrum (CD_3OD, 600 MHz) of **2**

Fig. S15. Comparison of ^{1}H NMR spectra of compounds **1** and **2** from δ_H 2.0 to 6.5 ppm.

Fig. S16. ^{13}C-NMR spectrum (CD_3OD, 150 MHz) of **2**

Fig. S17. ^{1}H-^{1}H COSY spectrum (CD_3OD) of **2**

Fig. S18. HSQC spectrum (CD_3OD) of **2**

Fig. S19. HMBC spectrum (CD_3OD) of **2**

Fig. S20. NOESY spectrum (CD_3OD) of **2**

MS, UV, IR and NMR Spectra of Compound 3

Fig. S21. HRESIMS spectrum of **3**

Fig. S22. IR spectrum of **3**

Fig. S23. UV spectrum in MeOH of **3**

Fig. S24. ^{1}H-NMR spectrum (CD_3OD, 600 MHz) of **3**

Fig. S25. ^{13}C-NMR spectrum (CD_3OD, 150 MHz) of **3**

Fig. S26. ^{1}H-^{1}H COSY spectrum (CD_3OD) of **3**

Fig. S27. HSQC spectrum (CD_3OD) of **3**

Fig. S28. HMBC spectrum (CD_3OD) of **3**

Fig. S29. NOESY spectrum (CD_3OD) of **3**

MS, UV, IR and NMR Spectra of Compound 1

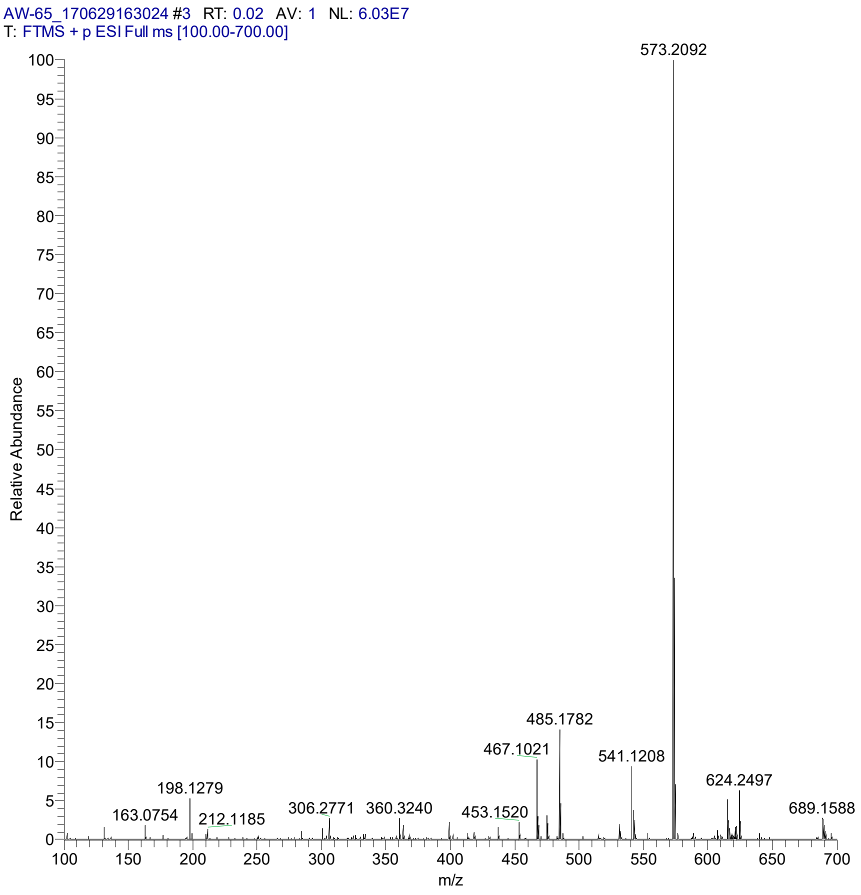

Fig. S1. HRESIMS spectrum of **1**

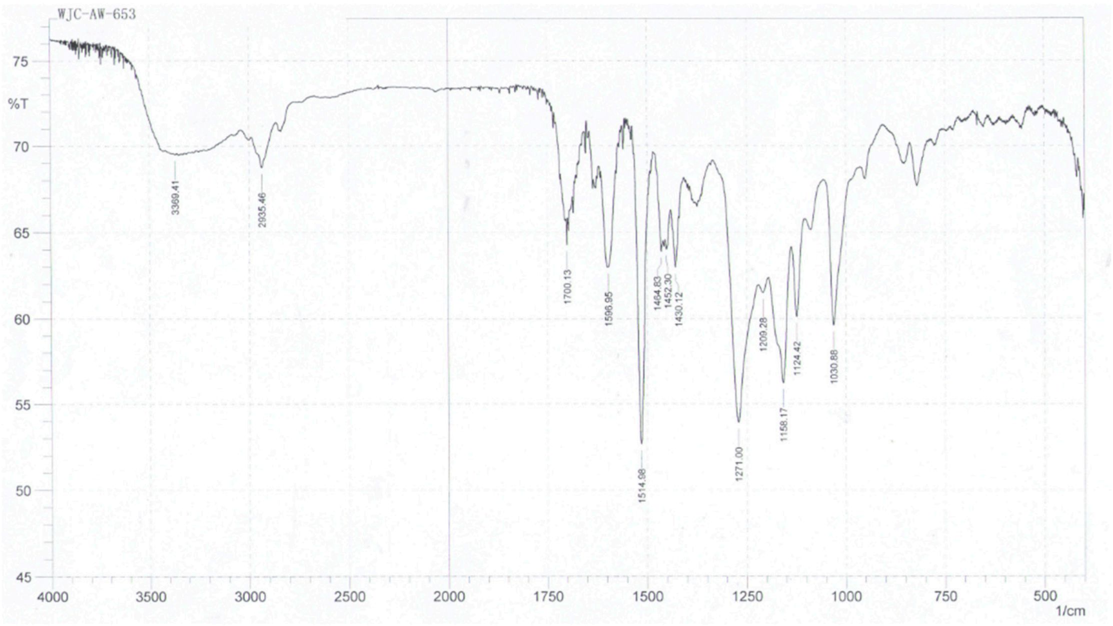

Fig. S2. IR spectrum of **1**

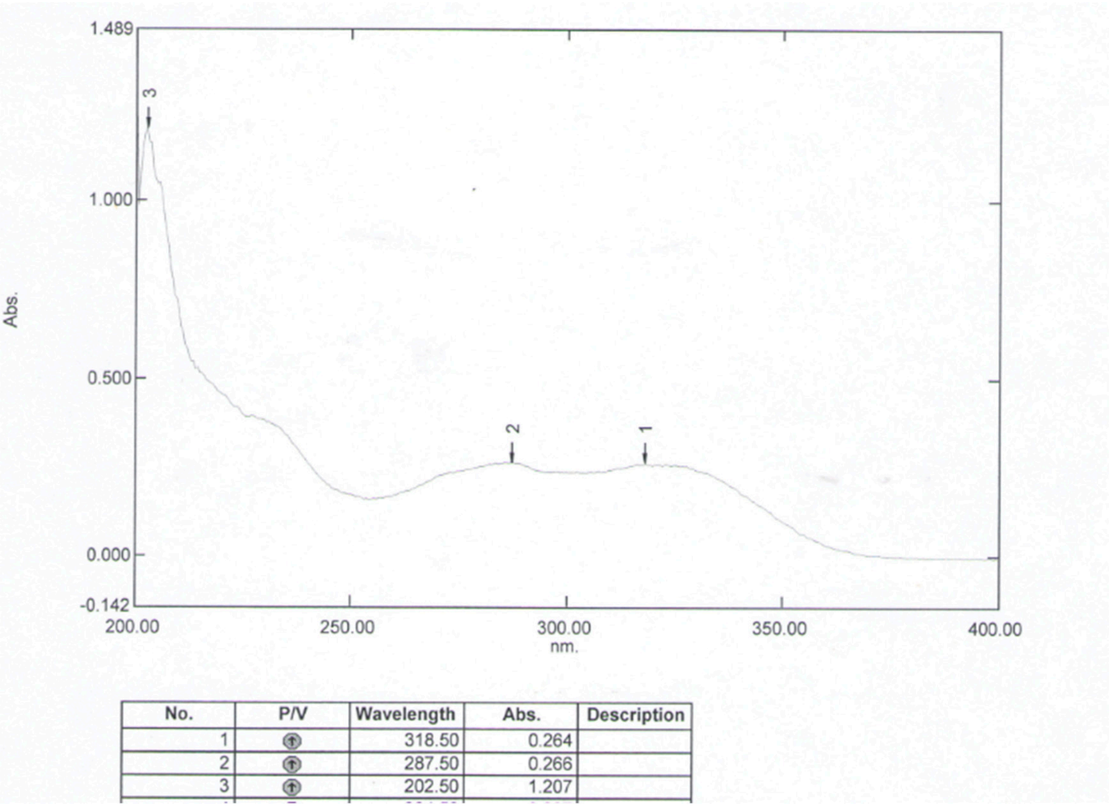

No.	P/V	Wavelength	Abs.	Description
1	⬆	318.50	0.264	
2	⬆	287.50	0.266	
3	⬆	202.50	1.207	

Fig. S3. UV spectrum in MeOH of **1**

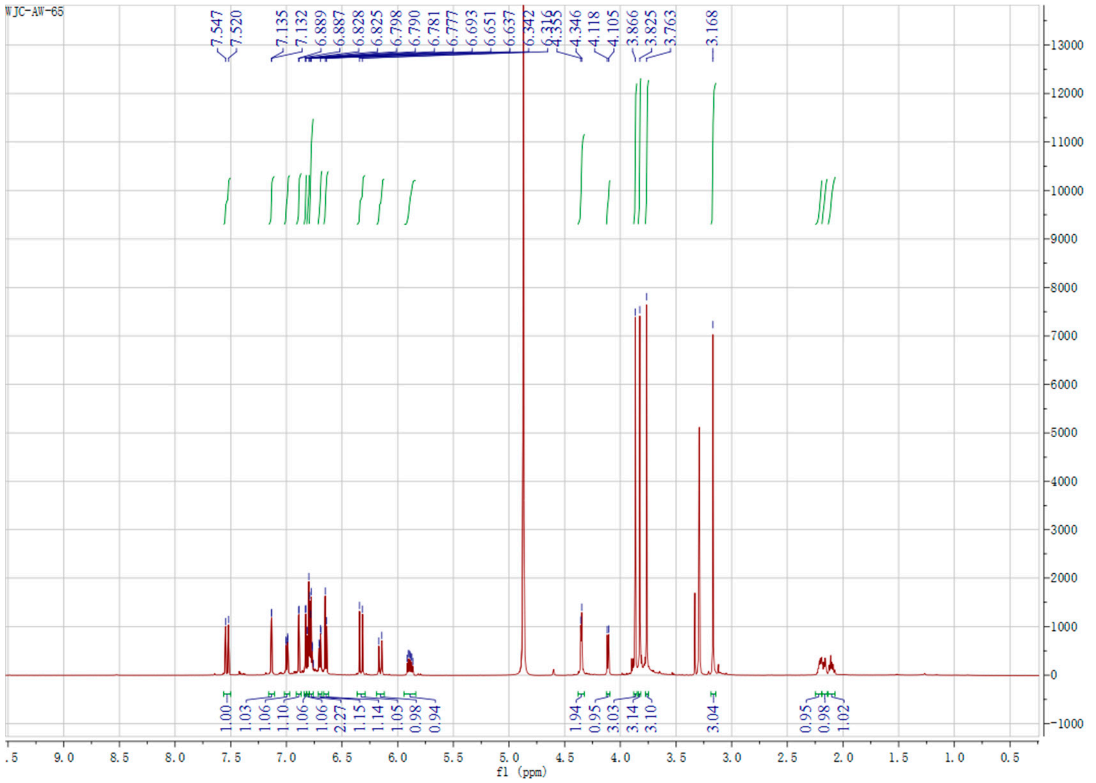

Fig. S4. ^{1}H-NMR spectrum (CD_3OD, 600 MHz) of **1**

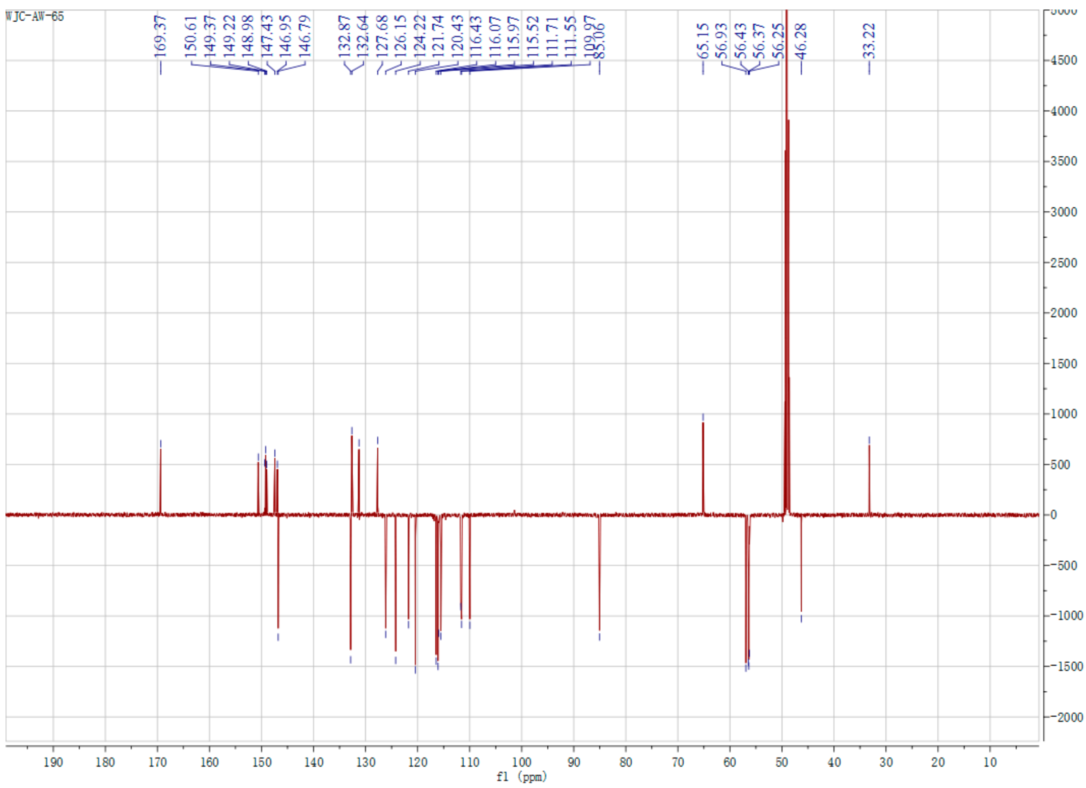

Fig. S5. ^{13}C-NMR spectrum (CD_3OD, 150 MHz) of **1**

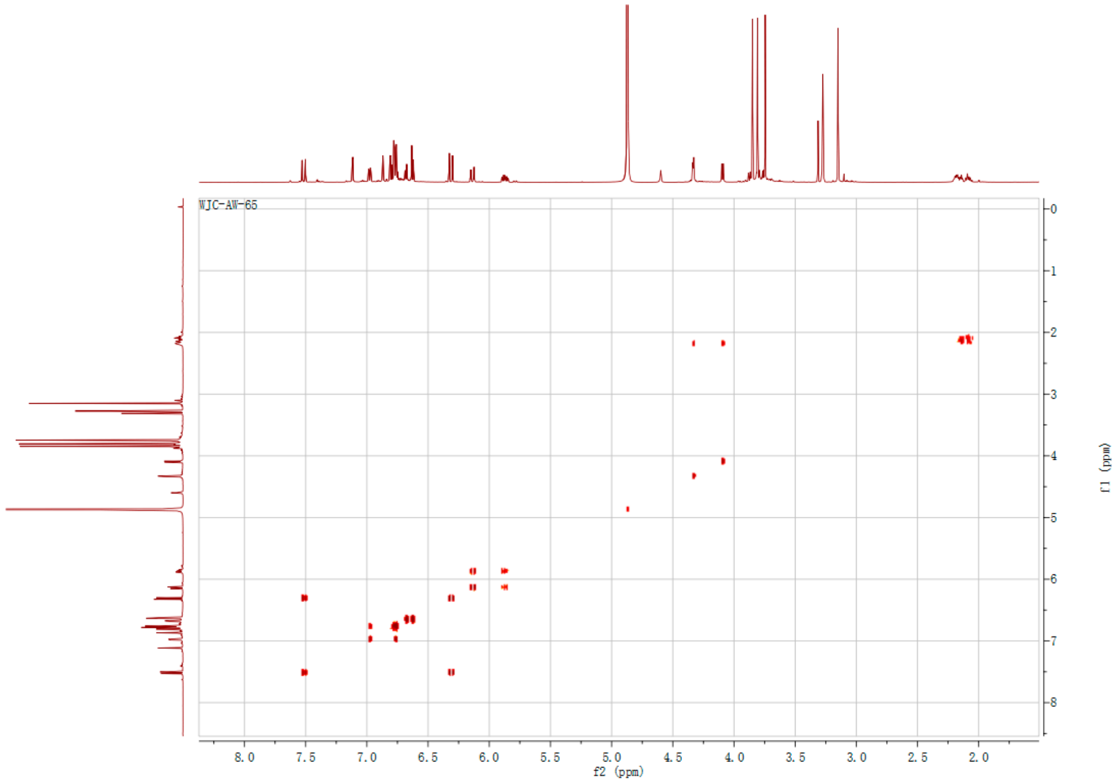

Fig. S6. ^{1}H-^{1}H COSY spectrum (CD_3OD) of **1**

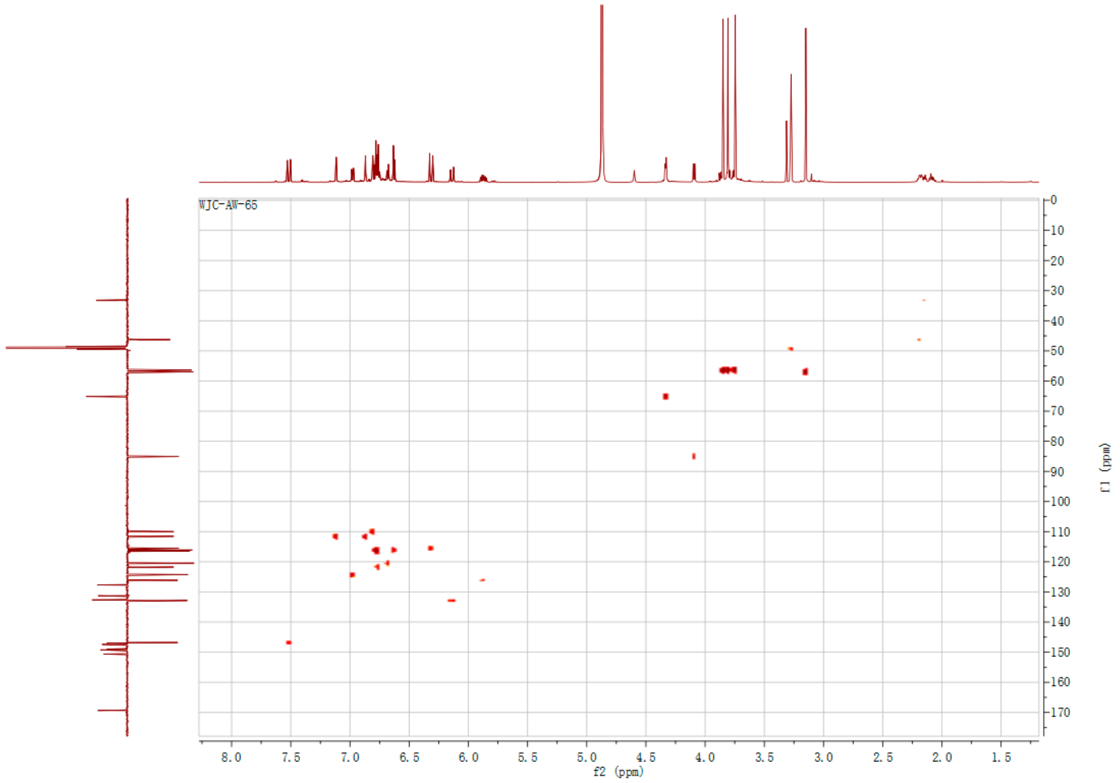

Fig. S7. HSQC spectrum (CD_3OD) of **1**

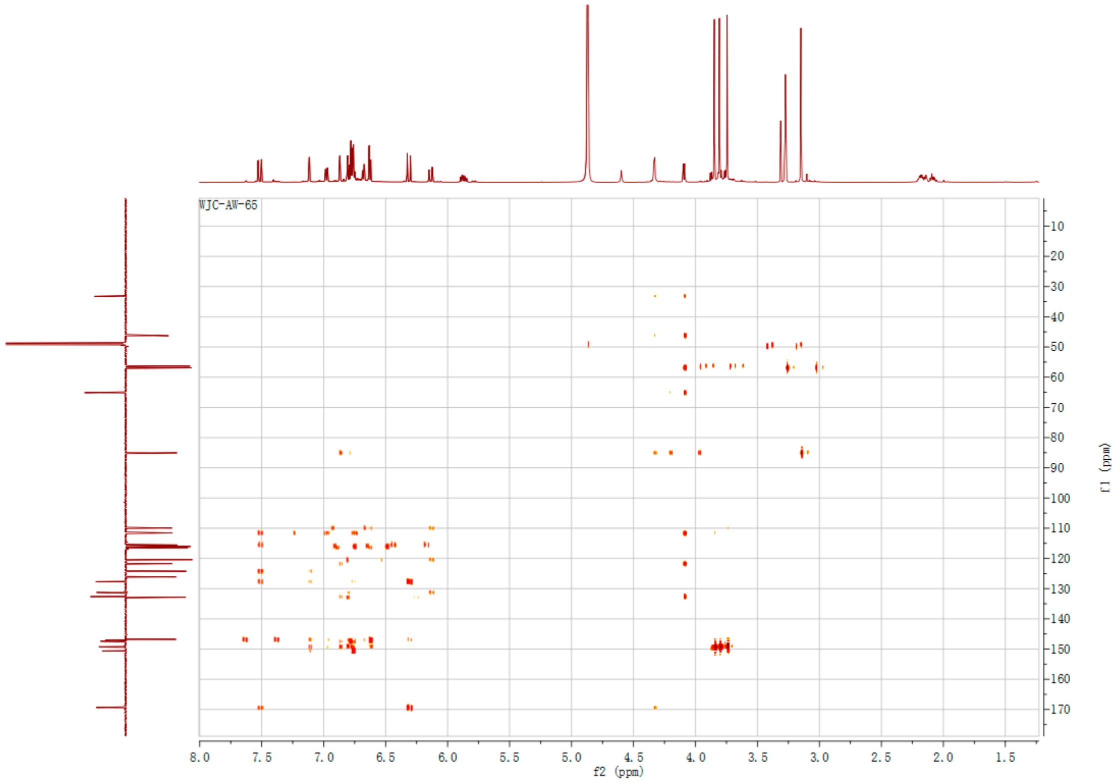

Fig. S8. HMBC spectrum (CD_3OD) of **1**

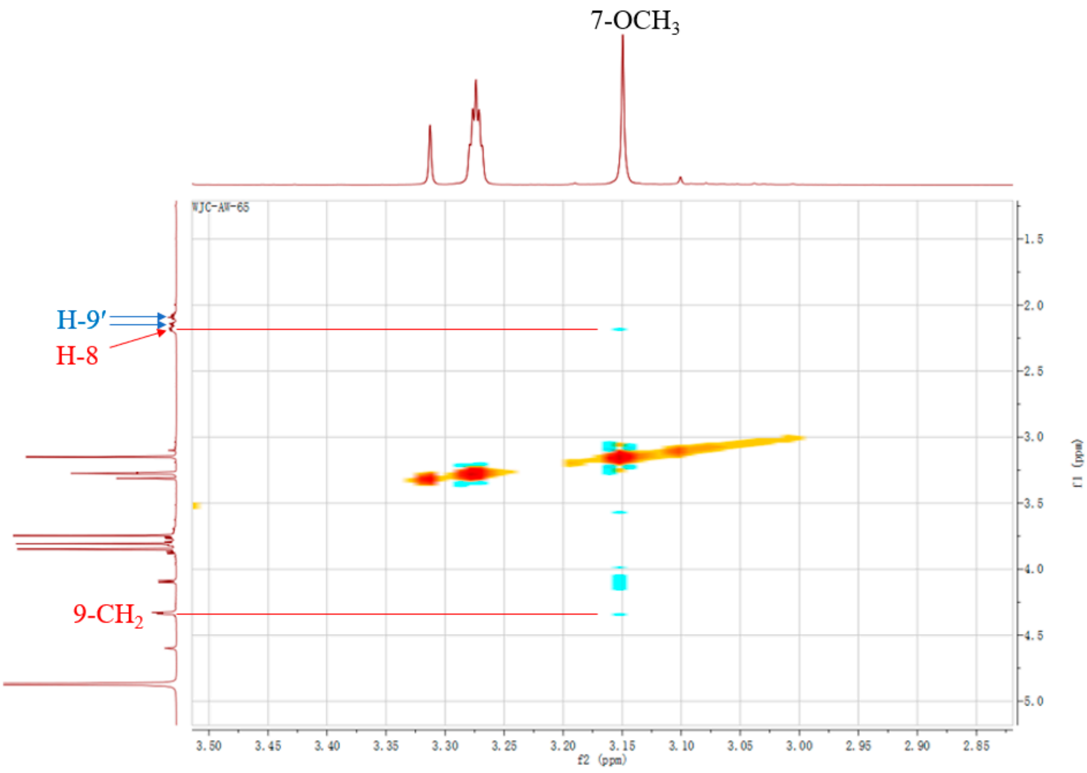

Fig. S9. NOESY spectrum (CD3OD) of **1**

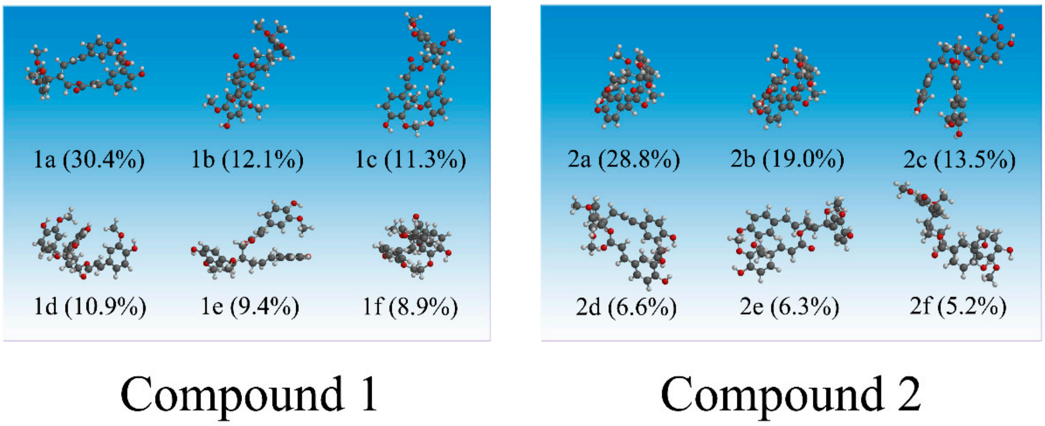

Fig. S10 The conformers of compounds **1** and **2** with a Boltzmann-population greater than 5% selected for ECD calculations

MS, UV, IR and NMR Spectra of Compound 2

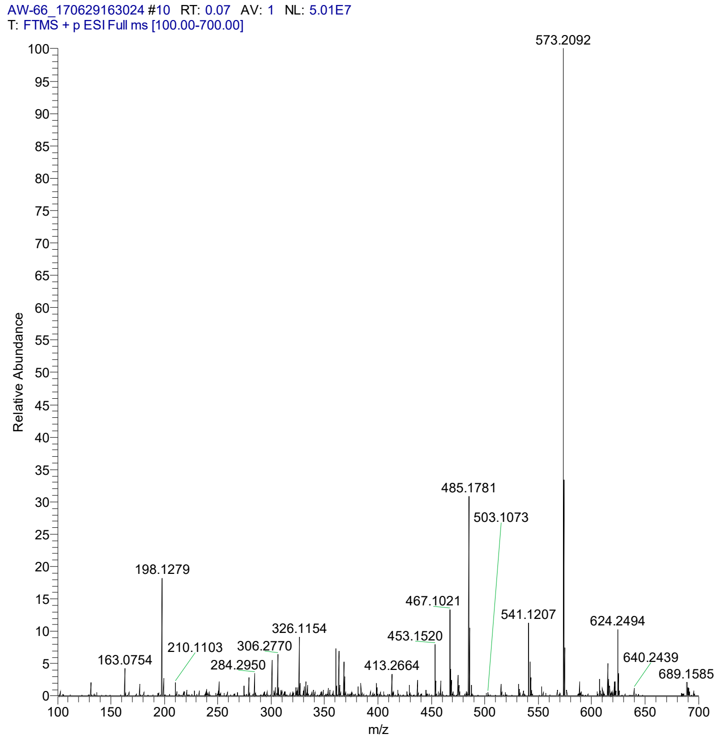

Fig. S11. HRESIMS spectrum of **2**

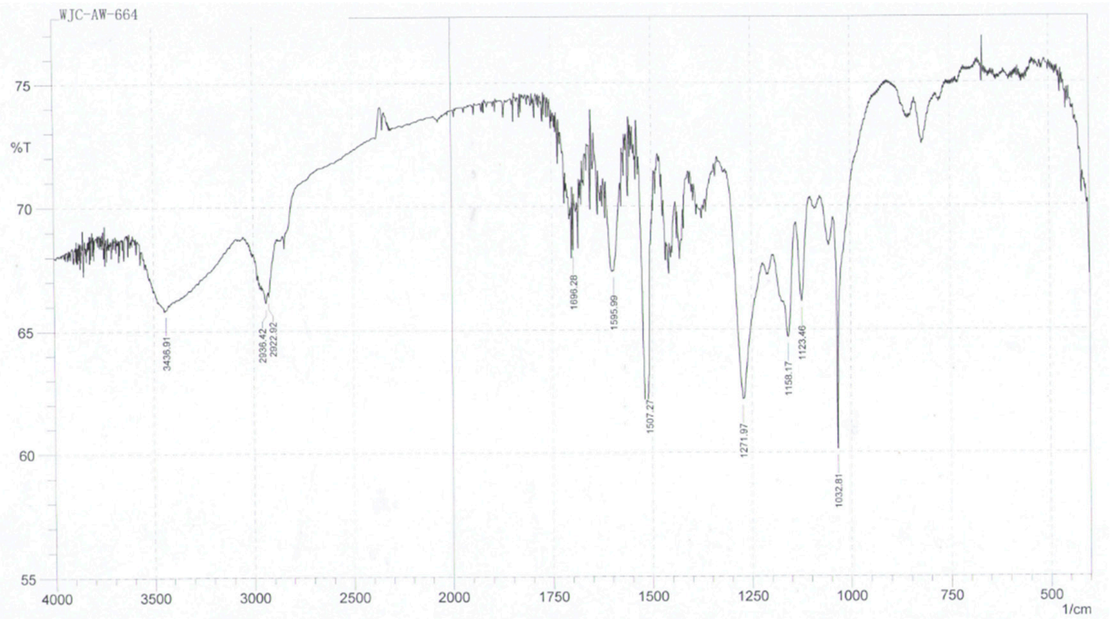

Fig. S12. IR spectrum of **2**

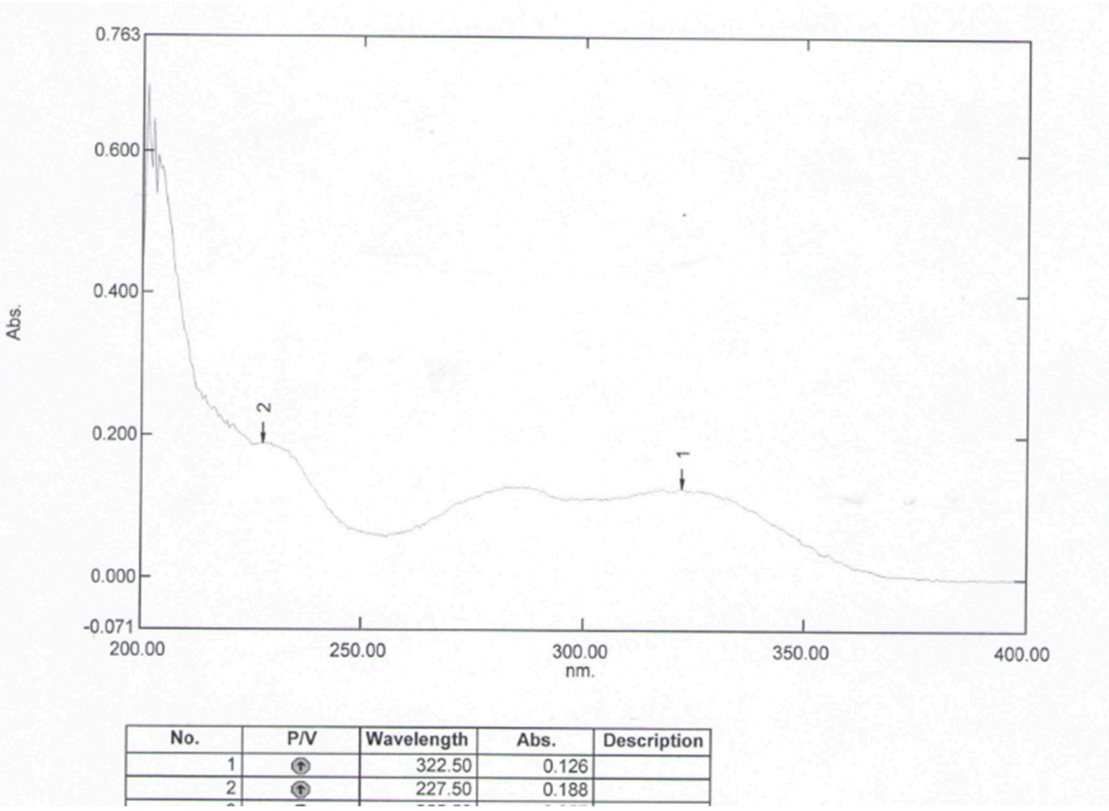

Fig. S13. UV spectrum in MeOH of **2**

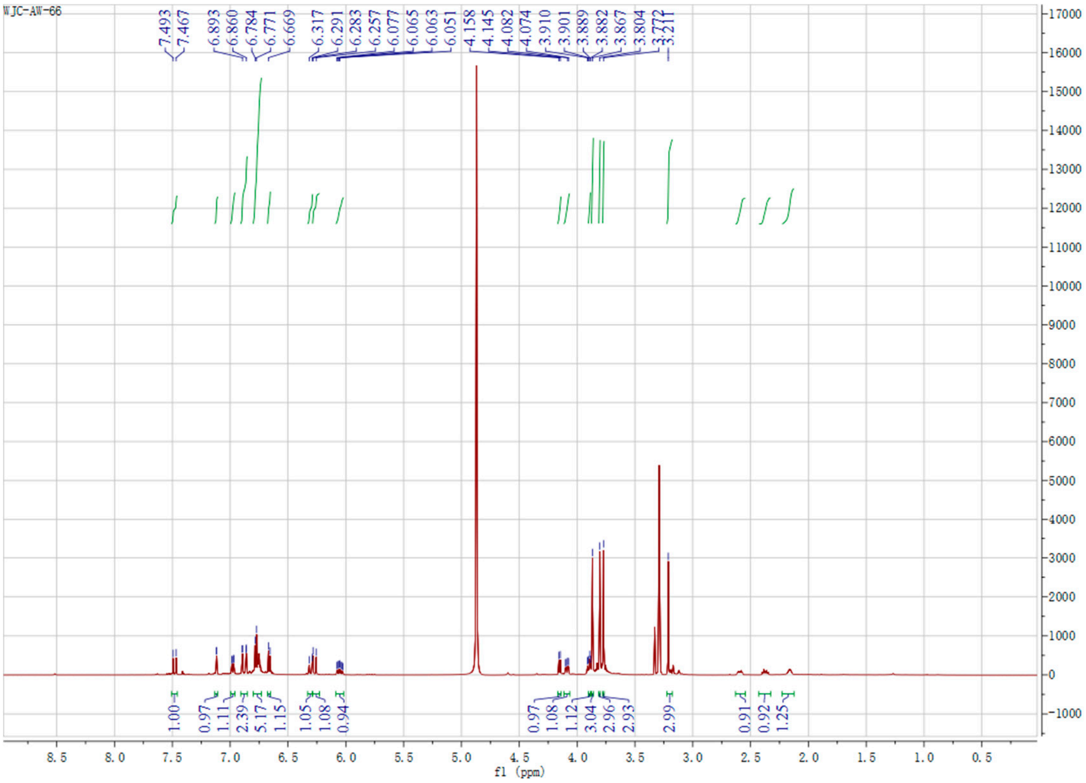

Fig. S14. ^{1}H-NMR spectrum (CD_3OD, 600 MHz) of **2**

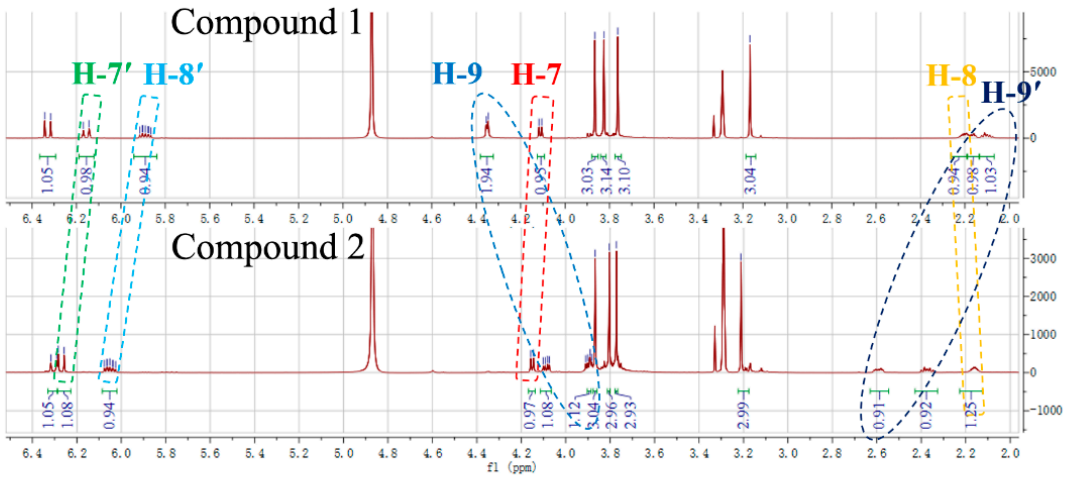

Fig. S15. Comparison of ^{1}H NMR spectra of compounds **1** and **2** from δ_H 2.0 to 6.5 ppm.

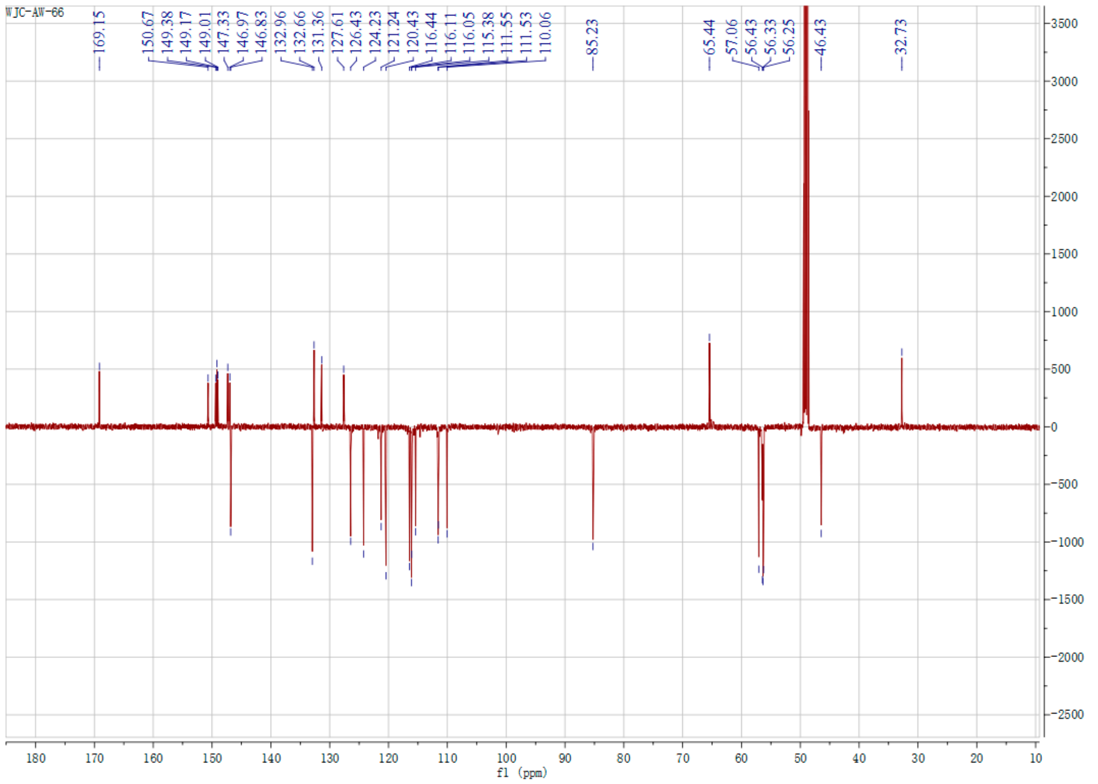

Fig. S16. ^{13}C-NMR spectrum (CD_3OD, 150 MHz) of **2**

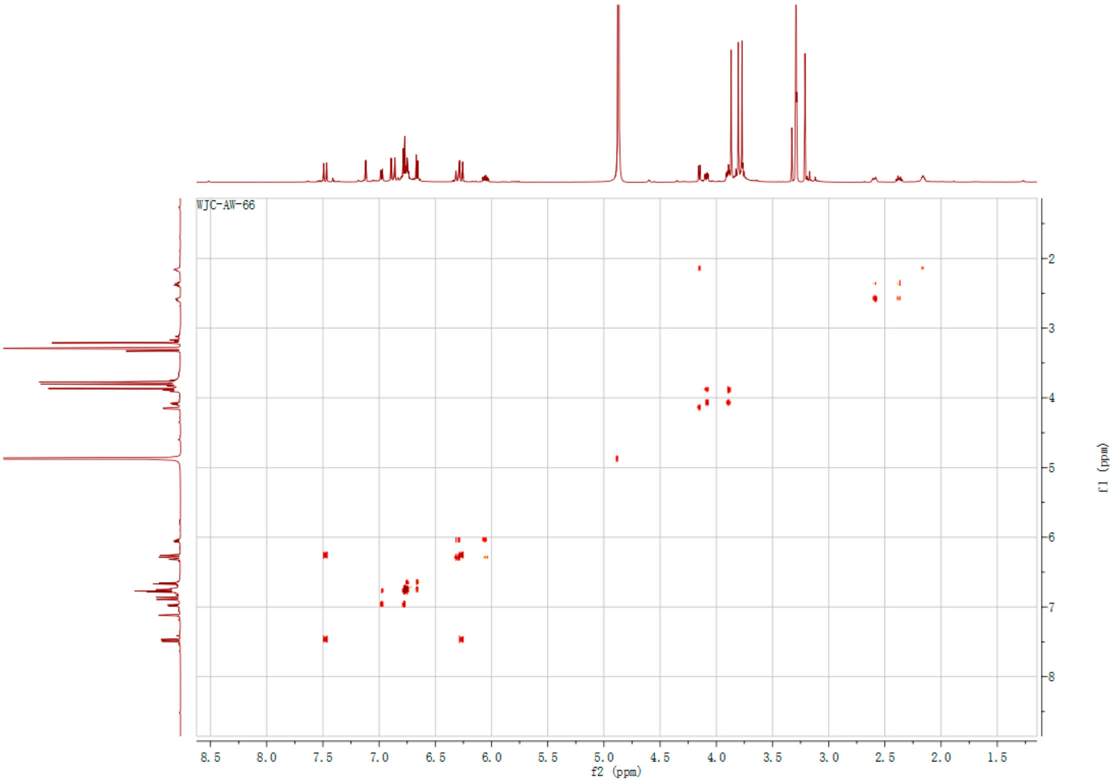

Fig. S17. ^{1}H-^{1}H COSY spectrum (CD_3OD) of **2**

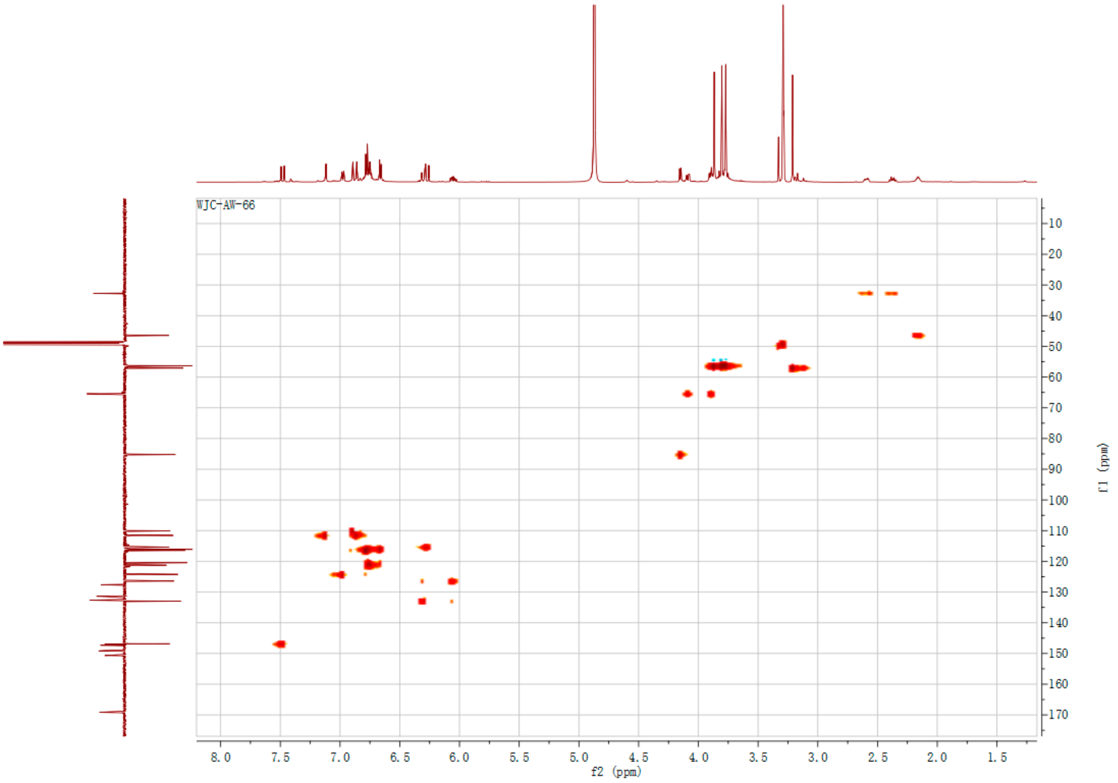

Fig. S18. HSQC spectrum (CD_3OD) of **2**

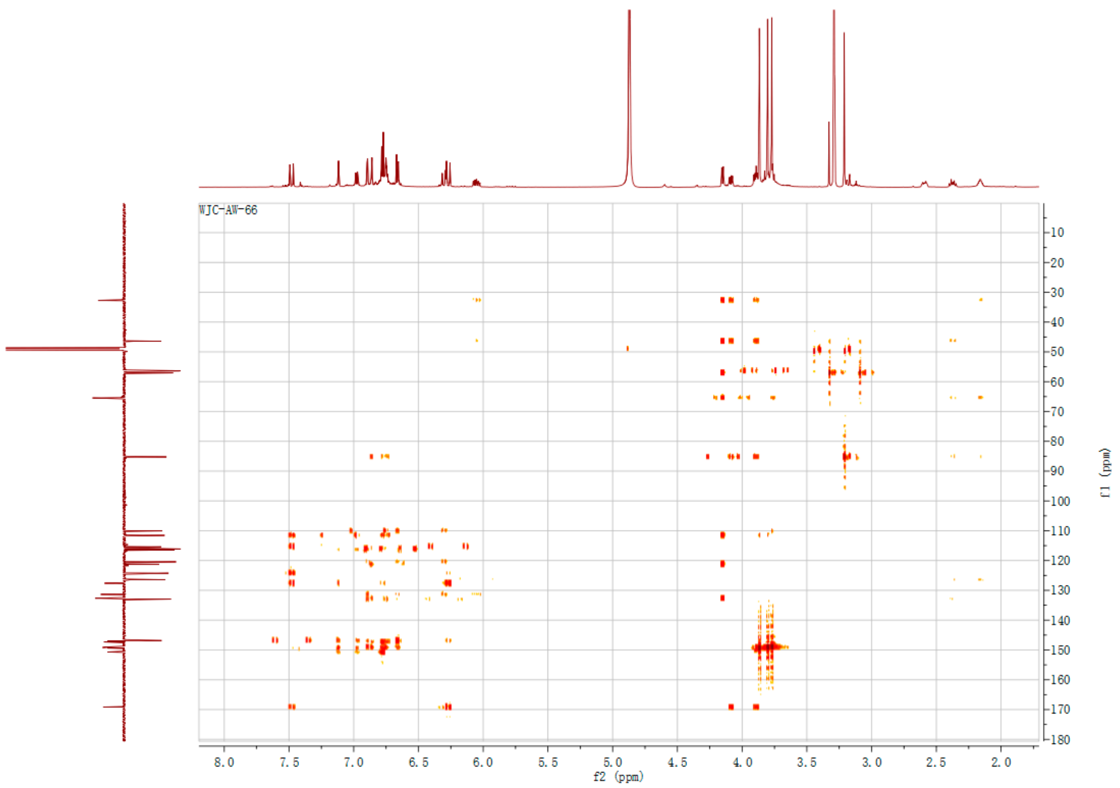

Fig. S19. HMBC spectrum (CD_3OD) of **2**

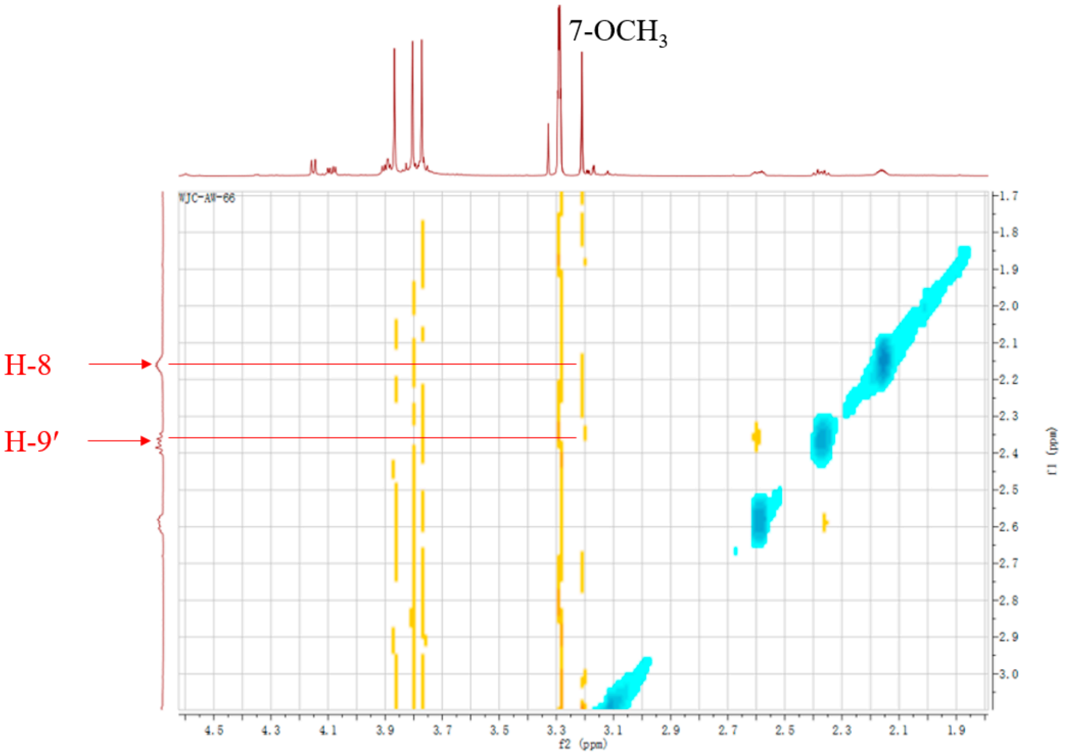

Fig. S20. NOESY spectrum (CD_3OD) of **2**

MS, UV, IR and NMR Spectra of Compound 3

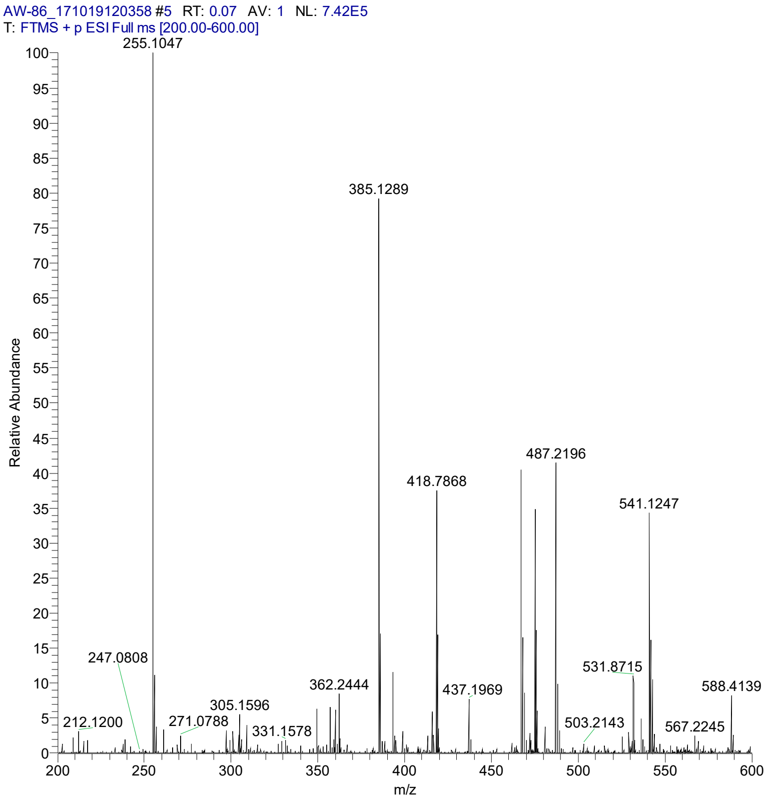

Fig. S21. HRESIMS spectrum of **3**

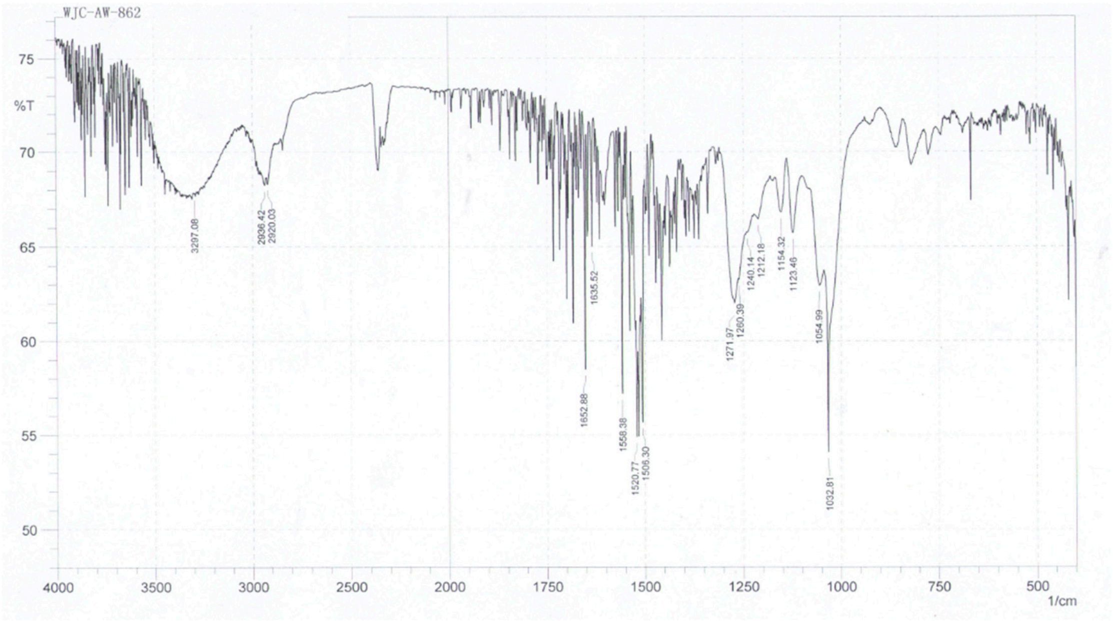

Fig. S22. IR spectrum of **3**

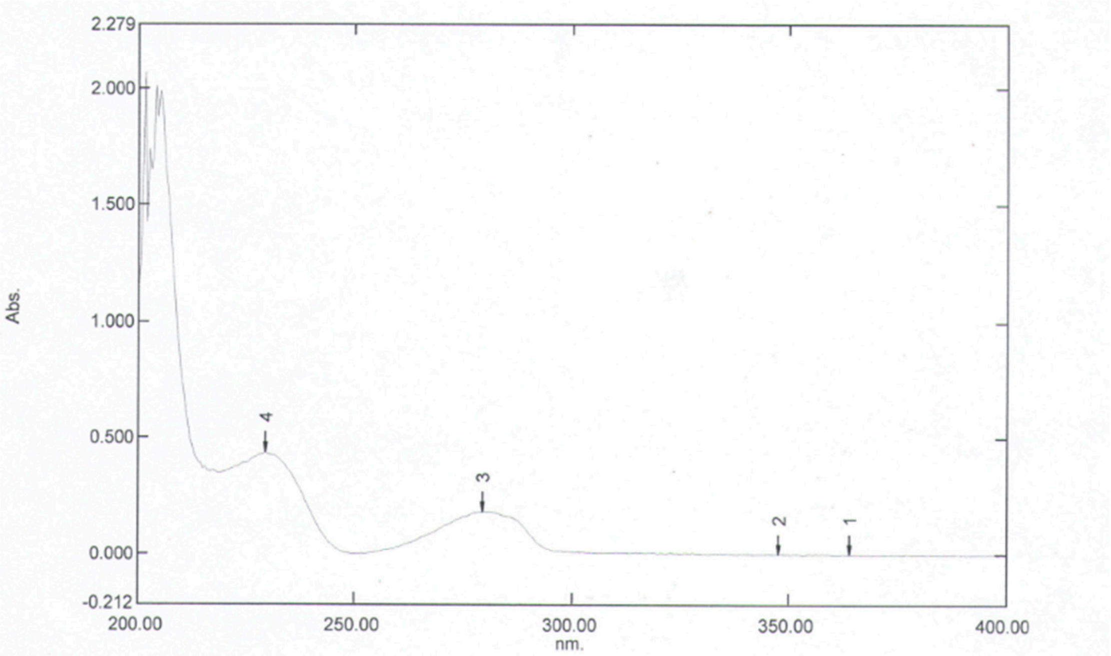

No.	P/V	Wavelength	Abs.	Description
1	⊕	364.00	-0.001	
2	⊕	348.00	0.002	
3	⊕	279.50	0.185	
4	⊕	229.50	0.438	

Fig. S23. UV spectrum in MeOH of **3**

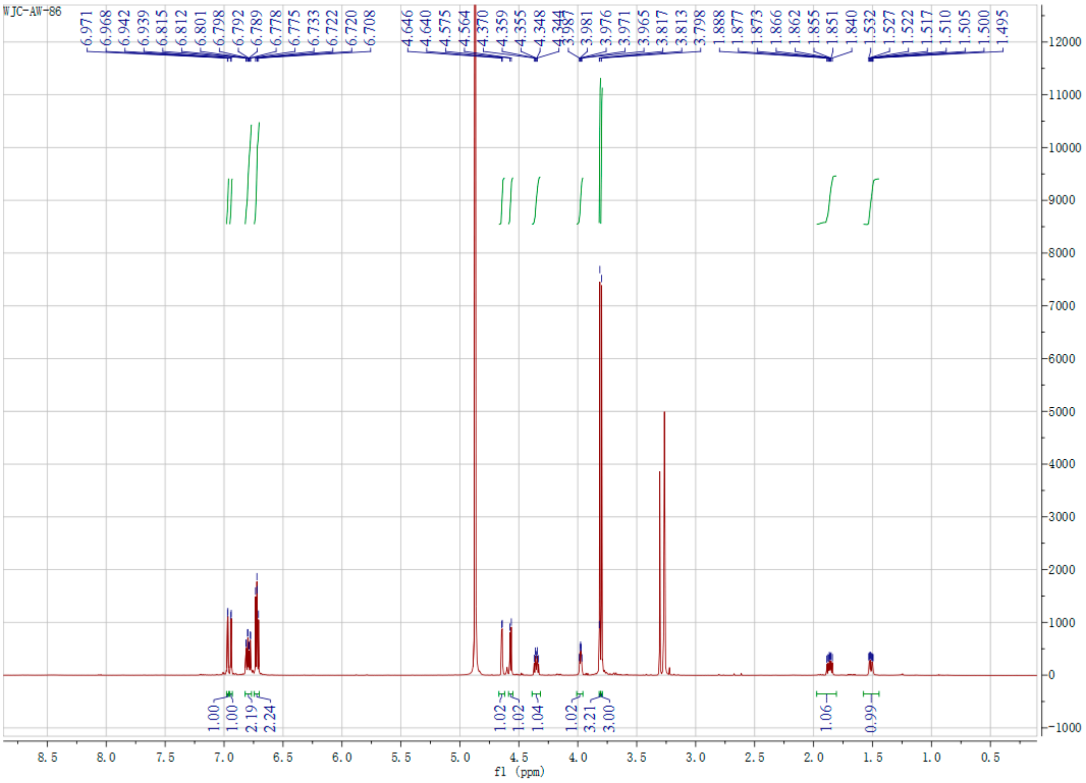

Fig. S24. ^{1}H-NMR spectrum (CD_3OD, 600 MHz) of **3**

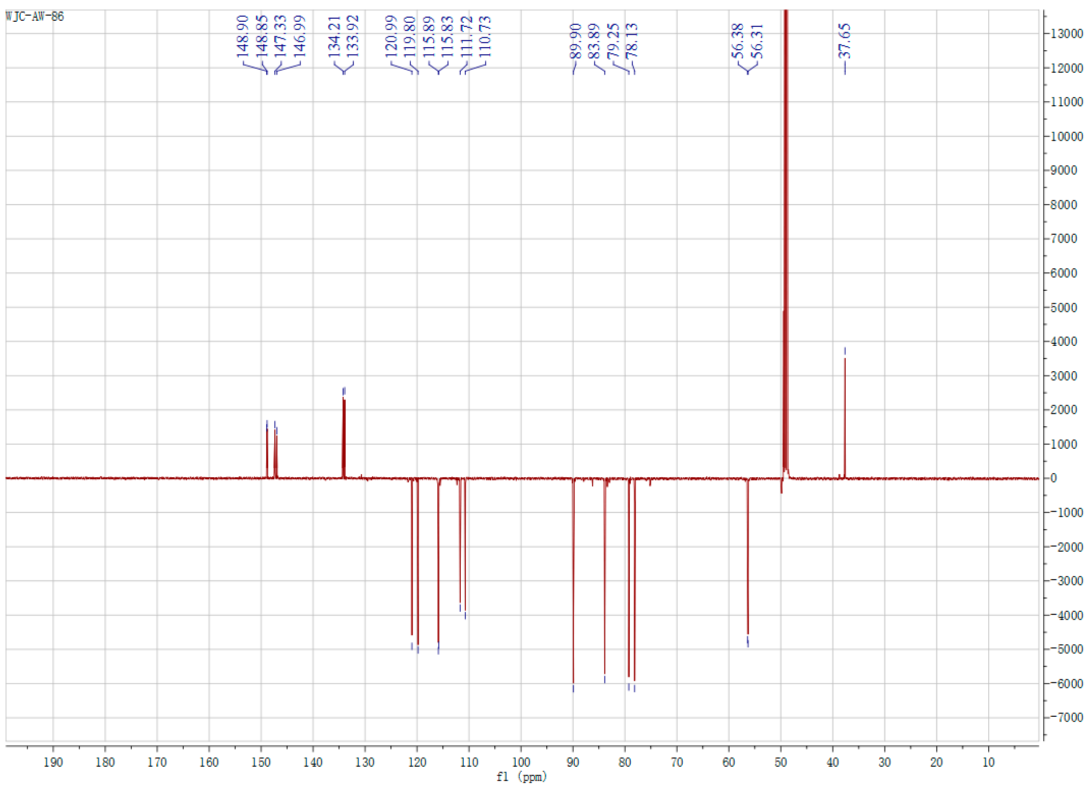

Fig. S25. ^{13}C-NMR spectrum (CD3OD, 150 MHz) of **3**

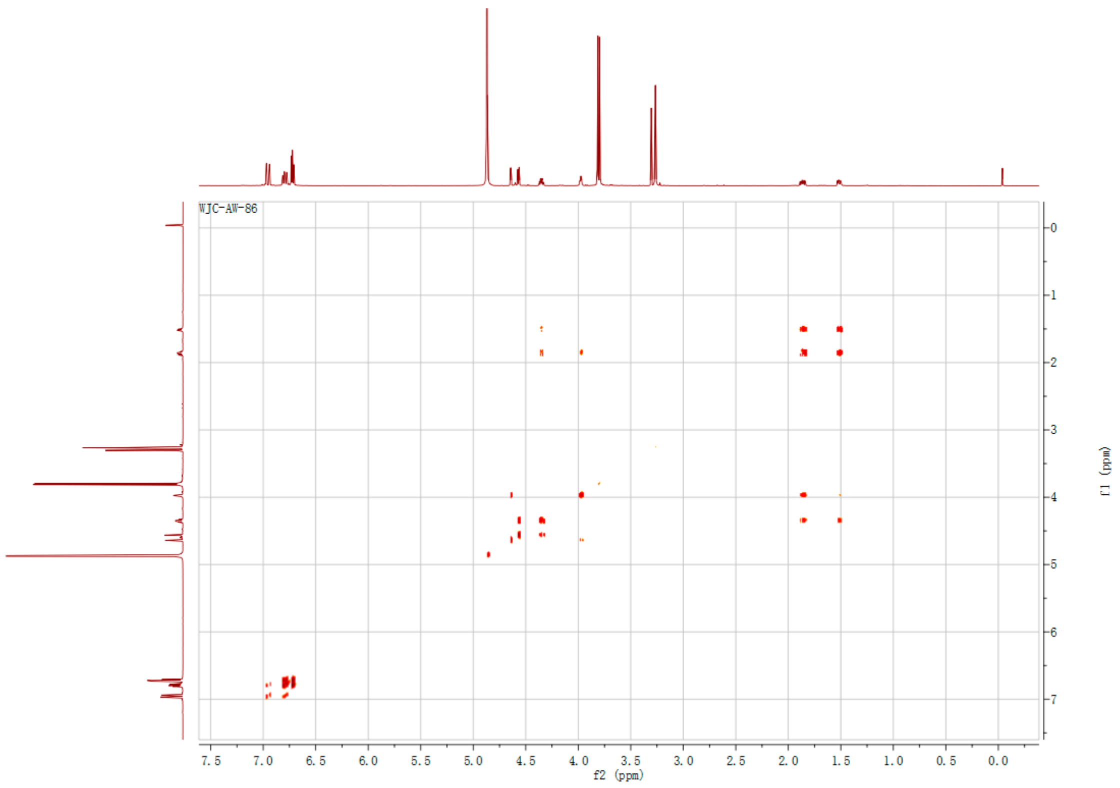

Fig. S26. ^{1}H-^{1}H COSY spectrum (CD3OD) of **3**

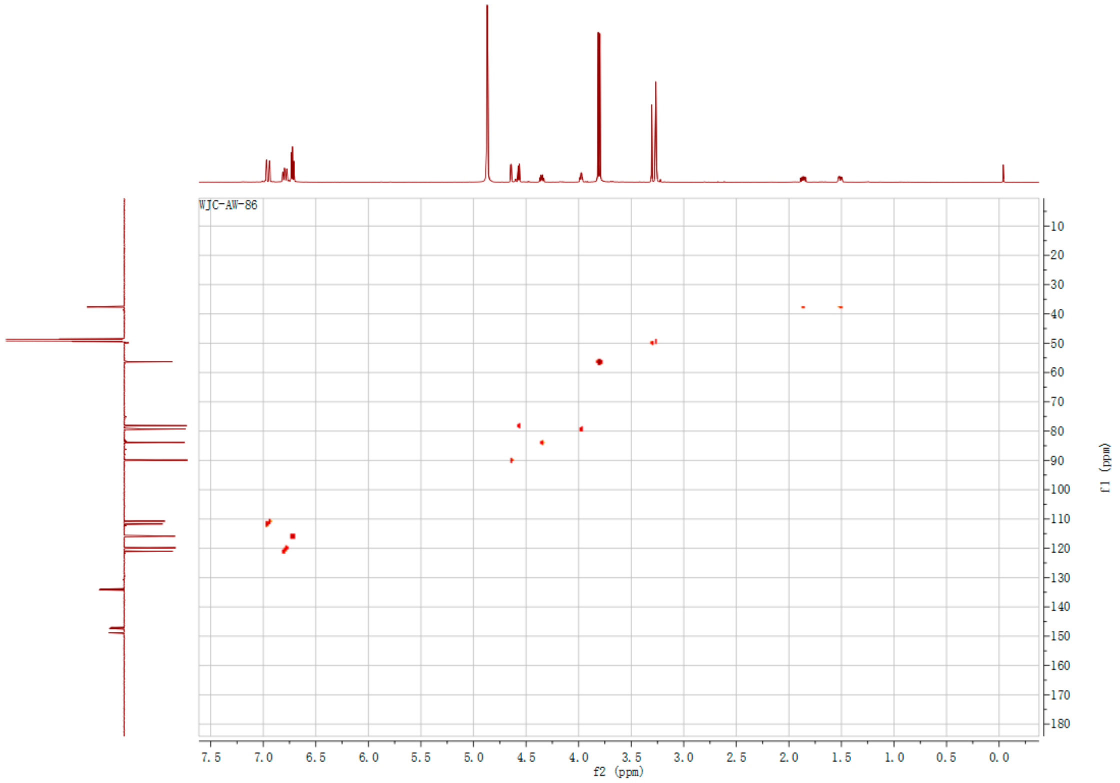

Fig. S27. HSQC spectrum (CD_3OD) of **3**

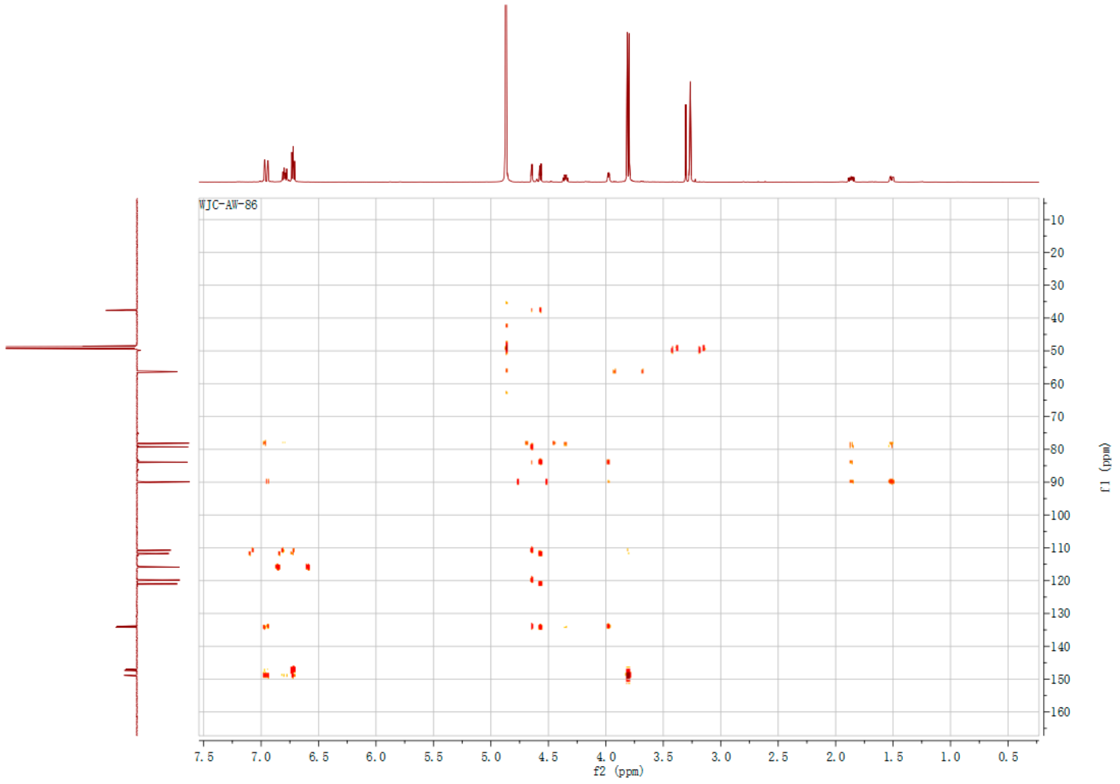

Fig. S28. HMBC spectrum (CD_3OD) of **3**

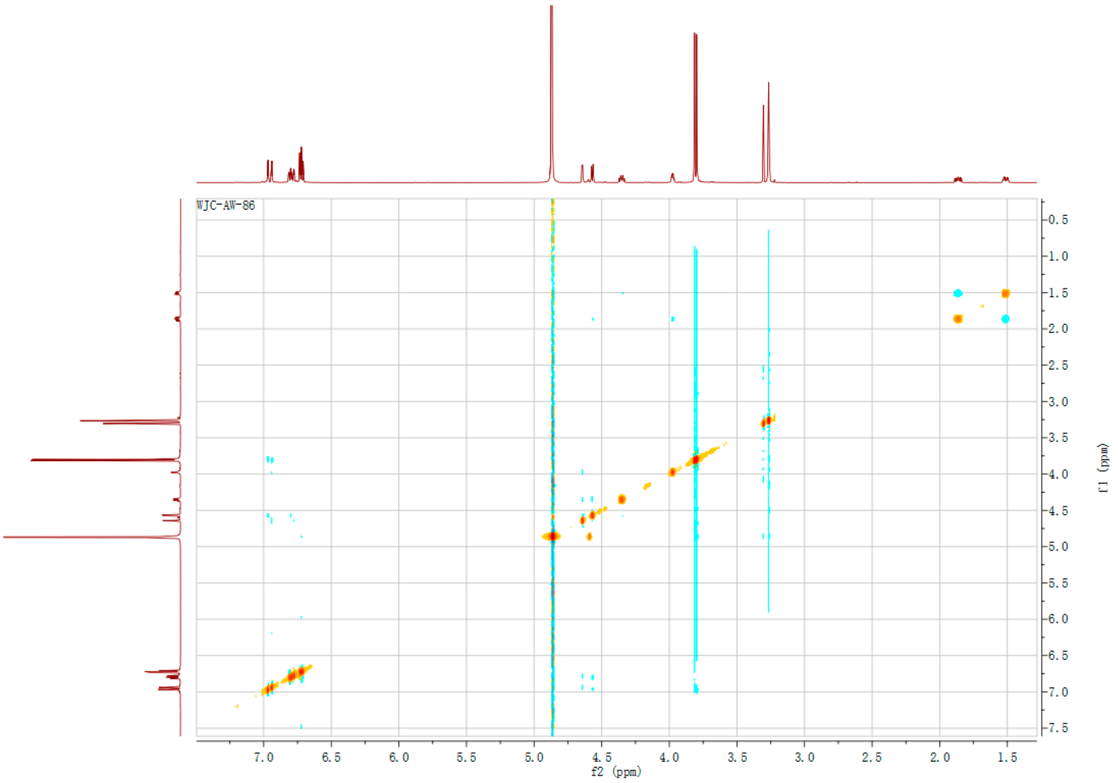

Fig. S29. NOESY spectrum (CD_3OD) of **3**
